# Effects of exercise on angiogenesis biomarkers in cancer patients: a systematic review and meta-analysis

**DOI:** 10.3389/fimmu.2025.1705472

**Published:** 2026-01-09

**Authors:** Jingyu Wang, Yuxuan He, Ziqian Wang, Zhouluo Wang, Yu Sun, Kyung-Hee Lee, Jae-Young Choi

**Affiliations:** 1 Department of Sport Leisure, Sungshin Women’s University, Seoul, Republic of Korea; 2 College of Education and Arts, Jiujiang Polytechnic University of Science and Technology, Jiujiang, China; 3 School of Philosophy and Sociology, Jilin University, Changchun, China; 4 Department of Exercise Prescription, Dongshin University, Jeollanam-do, Republic of Korea; 5 Department of Exercise Therapy, Gachon University, Seoul, Republic of Korea; 6 Department of Physical Education, College of Education, Korea University, Seoul, Republic of Korea

**Keywords:** angiogenesis, biomarkers, cancer, exercise, exercise prescription

## Abstract

**Objective:**

Angiogenesis plays a central role in tumor growth, progression, and treatment response. The primary aim of this systematic review and meta-analysis was to determine the effects of regular exercise on angiogenesis biomarkers in patients with cancer.

**Methods:**

Randomized controlled trials (RCTs) were searched across five databases up to July 2025. Eligible studies included adults with cancer (≥18 years), exercise interventions lasting more than four weeks, and at least one reported angiogenesis biomarker. The Cochrane risk of bias tool was used to assess RCT quality, and the Grading of Recommendations, Assessment, Development, and Evaluation (GRADE) approach was applied to evaluate evidence certainty. Meta-analysis and meta-regression were performed using robust variance estimation.

**Results:**

Thirteen RCTs were included. Pooled estimates suggested a small, nonsignificant difference in circulating VEGF favoring exercise (d = −0.14, p = 0.36). Similarly small, nonsignificant differences were observed for VCAM-1 (d = 0.24, p = 0.15) and MCP-1 (d = −0.20, p = 0.36).

**Conclusion:**

Regular exercise was associated with only small and nonsignificant changes in angiogenesis biomarkers in patients with cancer. Although these preliminary patterns suggest potential interactions with angiogenic and immune pathways, the low certainty of evidence limits firm conclusions. Future randomized controlled trials are needed to confirm these effects and clarify how exercise prescriptions at different treatment stages influence angiogenesis.

**Systematic review registration:**

https://www.crd.york.ac.uk/PROSPERO/, identifier CRD420251088092.

## 1 Introduction

Angiogenesis is an essential physiological process in human embryonic development and tissue repair, but it can be abnormally activated or disrupted in many diseases (1). In tumors, persistent hypoxia and the upregulation of pro-angiogenic factors drive abnormal angiogenic activation (2). As tumor volume increases and metabolic demands rise, angiogenesis not only sustains tumor growth but also promotes invasion, immune evasion, and distant metastasis through aberrant vascular structures (3). Compared with solely inhibiting angiogenesis, current research has shifted toward restoring vascular function to achieve vascular normalization, thereby slowing tumor progression and enhancing the efficacy of anticancer therapies (4).

Multiple molecules within the tumor microenvironment (TME) regulate angiogenesis, including growth factors such as vascular endothelial growth factor (VEGF), adhesion molecules, and matrix metalloproteinases (3). VEGF is one of the key drivers of tumor angiogenesis and metastasis and has become a primary target of anti-angiogenic cancer therapies (5, 6). It is markedly upregulated in many solid tumors and induces a disorganized and highly permeable vascular network that supports tumor growth and progression (7).

Based on this biological foundation, growing evidence has highlighted the clinical significance of VEGF in cancer risk, screening, and prognosis. For cancer risk, Mendelian randomization studies have suggested a causal relationship between circulating VEGF levels and the incidence of colorectal cancer and colon adenocarcinoma (8). For diagnostic purposes, systematic review evidence indicates that circulating VEGF shows moderate diagnostic accuracy for ovarian cancer (9). For prognosis, several systematic reviews and meta-analyses have shown that elevated circulating VEGF is closely associated with overall survival or disease-free survival in patients with colorectal cancer, lung cancer, intrahepatic cholangiocarcinoma, ovarian cancer, breast cancer, and hepatocellular carcinoma (10–15).

In the therapeutic context, VEGF has gradually evolved from a pro-angiogenic factor to a potentially actionable biomarker with predictive value. Emerging phase III trial data suggest that serum VEGF-A and its isoforms may help identify patients with metastatic non-squamous non-small-cell lung cancer who could benefit from bevacizumab combined with immunotherapy (16). Similarly, in breast cancer, VEGF levels have been proposed as a candidate biomarker for bevacizumab treatment (17). Across cancer types, evidence shows that adding VEGF inhibitors to standard regimens can improve survival outcomes in patients with liver metastases (18). Broader data also indicate that anti-VEGF/VEGFR therapy combined with chemotherapy can improve progression-free survival and response rates in ovarian, breast, and prostate cancers, although with some increase in adverse events (19–21). Notably, some studies have reported that the clinical implications of elevated VEGF expression may also be related to paraneoplastic manifestations (22–24). Overall, VEGF possesses a dual role as both a biological driver and a clinically relevant biomarker.Growing evidence indicates that exercise, as a complementary therapy for cancer patients, can enhance the efficacy of anticancer treatments and reduce treatment-related adverse events (25). Exercise also reprograms the TME by reshaping metabolic, immune, and vascular systems (26, 27). In terms of angiogenesis, exercise promotes vascular normalization through mechanisms such as increased pericyte coverage and enhanced vascular maturity (26, 28). Two meta-analysis based on tumor animal models suggests that exercise may improve tumor vascularization (29, 30). Another meta-analysis in cancer patients reported that exercise improved endothelial function in breast and prostate cancer survivors (31). However, most existing systematic reviews have focused on inflammatory biomarkers such as IL-6, CRP, and TNF-α, while few have provided quantitative analyses centered on angiogenesis biomarkers. Only one meta-analysis in cancer patients included VEGF as an inflammatory marker, but it did not provide an in-depth interpretation of changes in VEGF (32). Therefore, the effects of exercise on angiogenesis biomarkers in patients with cancer remain insufficiently understood.

Therefore, this study aimed to include RCTs that reported exercise interventions of at least four weeks in cancer patients to systematically evaluate the effects of regular exercise on angiogenesis biomarkers. VEGF was the primary outcome, other angiogenesis biomarkers as secondary outcomes. Meta-analysis and meta-regression were conducted using the robust variance estimation (RVE) method. Compared with traditional approaches, RVE is better suited for handling the dependency of multiple correlated effect sizes reported within the same study and remains robust under small-sample conditions (33, 34). The specific objectives were (1): to synthesize effect sizes using RVE to determine the impact of exercise interventions on angiogenesis biomarkers in cancer patients; and (2) to conduct prespecified univariable RVE meta-regressions on exercise-related moderators to explore the potential moderating effects of intervention characteristics on angiogenesis biomarkers.

## 2 Methods

This systematic review was registered in PROSPERO (registration number: CRD420251088092) and conducted in accordance with the PRISMA guidelines (**Supplementary Material 1**) (35).

### 2.1 Data sources, literature search, and eligibility criteria

A systematic search of five databases was performed from their inception to July 6, 2025: PubMed (MEDLINE), Embase, the Cochrane Central Register of Controlled Trials (CENTRAL), SPORTDiscus, and Web of Science. In addition, reference lists of included articles and prior systematic reviews or meta-analyses were screened to identify relevant studies. The search strategy was developed based on the PICOS framework (**Supplementary Material 2**).

The inclusion criteria were as follows: population, cancer patients aged 18 years or older; intervention, exercise interventions lasting at least four weeks, including weight loss programs with explicitly reported exercise prescription parameters; comparator, non-exercise control groups, including usual care, health education, or waitlist controls; outcomes, at least one circulating angiogenesis-related biomarker; study design, randomized controlled trials (RCTs).

The exclusion criteria were as follows: population, cancer patients younger than 18 years; intervention, rehabilitation training, mind-body exercise such as yoga or tai chi, and exergames; comparator, no non-exercise control group; outcomes, biomarkers primarily representing systemic inflammation such as IL-6 and IL-8, and studies that did not report circulating angiogenesis biomarkers; study design, systematic reviews, narrative reviews, animal studies, and non-English publications. Studies were also excluded if the full text or relevant data could not be obtained after contacting the authors.

We *a priori* excluded mind–body exercise (e.g., yoga, tai chi), exergames, and rehabilitation programs. These modalities combine low-intensity activity with substantial meditative or cognitive components, leading to biological mechanisms and dose characteristics that differ from structured aerobic and resistance training. Existing evidence also suggests that mind–body exercise may have limited and uncertain effects on circulating inflammatory biomarkers in cancer populations (36). In addition, their wide variability in movement patterns, device use, and training dose makes key exercise parameters difficult to harmonize for quantitative synthesis. To preserve conceptual and methodological consistency and to align with guideline-based exercise prescriptions, this review focused on conventional aerobic and resistance training.

### 2.2 Study selection and data extraction

Two authors (YX H and ZQ W) independently reviewed relevant studies to identify potentially eligible trials that met the inclusion criteria. Disagreements were resolved by a third senior author (JY W). The following study characteristics were extracted: basic study information (authors, registration number, etc.), outcome measures (excluding follow-up), and moderators. For studies that reported data in formats other than mean ± SD (such as median, interquartile range, or standard error), data were converted according to the Cochrane Handbook for Systematic Reviews of Interventions (37).

### 2.3 Risk of bias and quality assessment

The Cochrane risk-of-bias tool (RoB 2) was used to assess the risk of bias in the included RCTs (38). Two authors (YX H and ZQ W) independently conducted the assessment, and disagreements were resolved by a third author (JY W). Inter-rater agreement reached an acceptable level (Kappa = 0.91). Given the nature of exercise interventions, “blinding of participants and personnel” was considered low risk. The certainty of evidence was assessed using the GRADE (Grading of Recommendations, Assessment, Development, and Evaluation) framework (39).

### 2.4 Data analysis

All statistical analyses were performed using R (version 4.4.1). Cohen’s d and its variance were used to pool effect sizes. A d value of 0.2 indicated a small effect, 0.5 a medium effect, and 0.8 a large effect (40). When trials reported multiple relevant comparisons (e.g., combined vs. control, aerobic vs. control) or multiple biomarkers within the same construct, we derived separate effect sizes for each. The RVE method was applied using the “robumeta” package. Regression models adopted a correlated effects structure, assuming within-study correlation of 0.5 (model weight = “CORR,” rho = 0.5). The rho parameter represents the assumed correlation among effect sizes derived from the same study and is required to model within-study dependence in the RVE framework. An RVE meta-regression model without covariates was used to estimate the overall mean effect size. Sensitivity analyses were conducted by varying rho from 0.1 to 0.9 in increments of 0.1 (e.g., rho = 0.1, 0.2). Between-study heterogeneity was assessed using the I² statistic, with values above 25% indicating low heterogeneity, above 50% moderate heterogeneity, and above 75% high heterogeneity (41). Twelve potential moderators, including exercise type and session intensity, were tested in univariable RVE meta-regression models, with I² and R² recorded for each model. In RVE models, R² represents the proportion of heterogeneity explained by the moderator, and the model with an R² closest to 1 was considered optimal (42). Publication bias was examined using Egger’s test in the “metafor” package, and the trim-and-fill method was applied to adjust for potential bias and generate corrected funnel plots. Data visualization was performed using the “ggplot2” package.

## 3 Results

**Figure 1:**
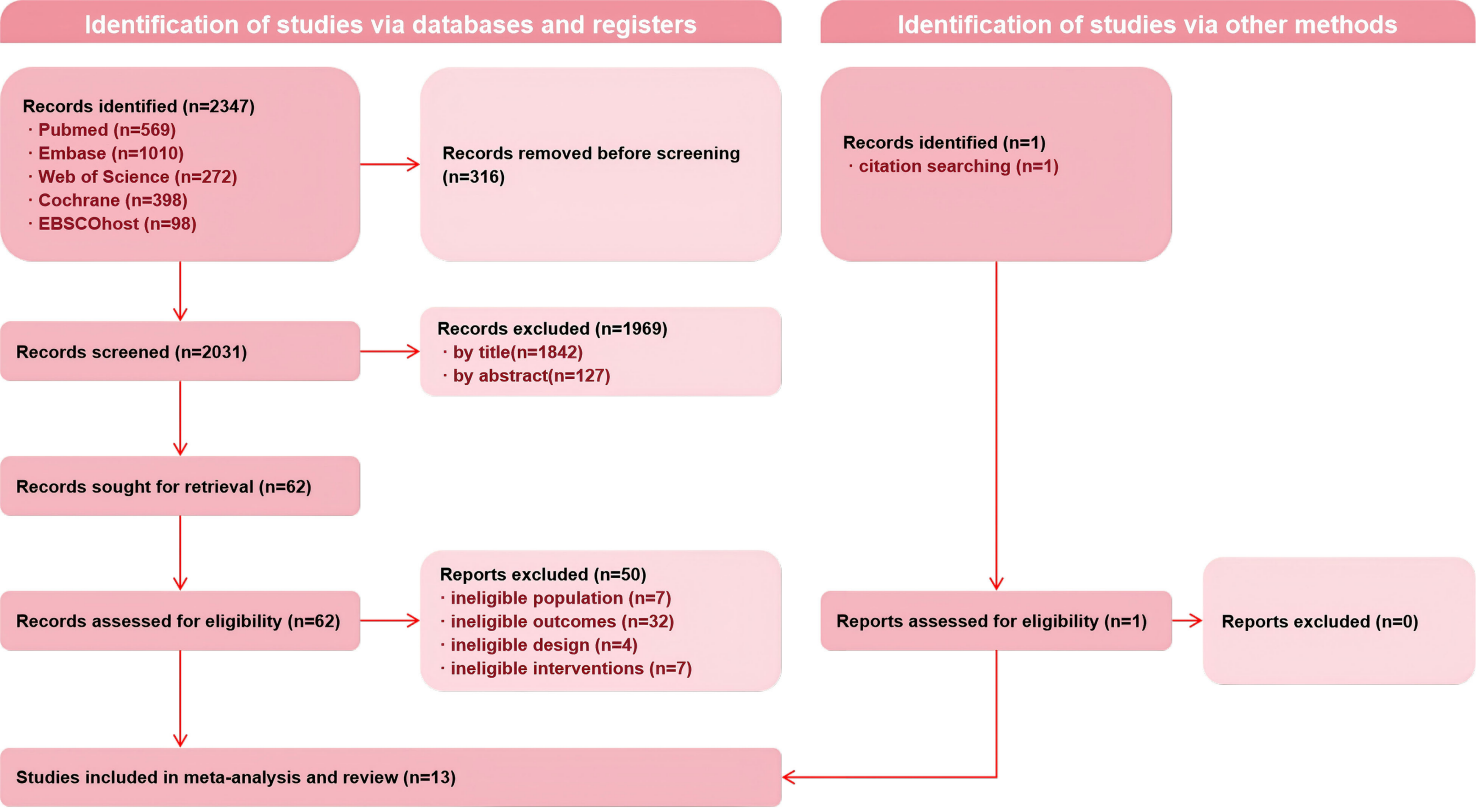
Preferred reporting items for systematic reviews and meta-analyses (PRISMA) flow diagram of record identification, screening, and selection processes.

A total of 2347 abstracts were initially identified, and data from 13 RCTs were finally included. The complete screening and selection process is shown in **Figure 1**.

### 3.1 Overview of included studies and meta-analysis results

Details of the included RCTs and reported outcomes are provided in (**Supplementary Material 3**). Independent effect sizes were extracted for RCTs with multiple intervention arms. Meta-analysis and meta-regression were conducted for VEGF, while only meta-analyses were performed for ICAM-1, VCAM-1, MCP-1, and VEGF-C. Due to the small number of RCTs (≤3) reporting other angiogenesis biomarkers such as FGF and PlGF, no meta-analyses were performed for these outcomes. (**Supplementary Material 4**) (**Supplementary Table S1**) reports the RVE model parameters. **Supplementary Material 4** (**Supplementary Table S2**) presents the results of a sensitivity analysis in which rho was varied from 0.1 to 0.9 in increments of 0.1, showing that changes in rho did not affect the model results. One RCT reported exercise-related adverse events, mainly pain (joint pain, back pain, and muscle pain), flu-like symptoms, and foot blisters (43). No study reported serious exercise-related adverse events.

#### 3.1.1 Effects of exercise on VEGF

**Figure 2:**
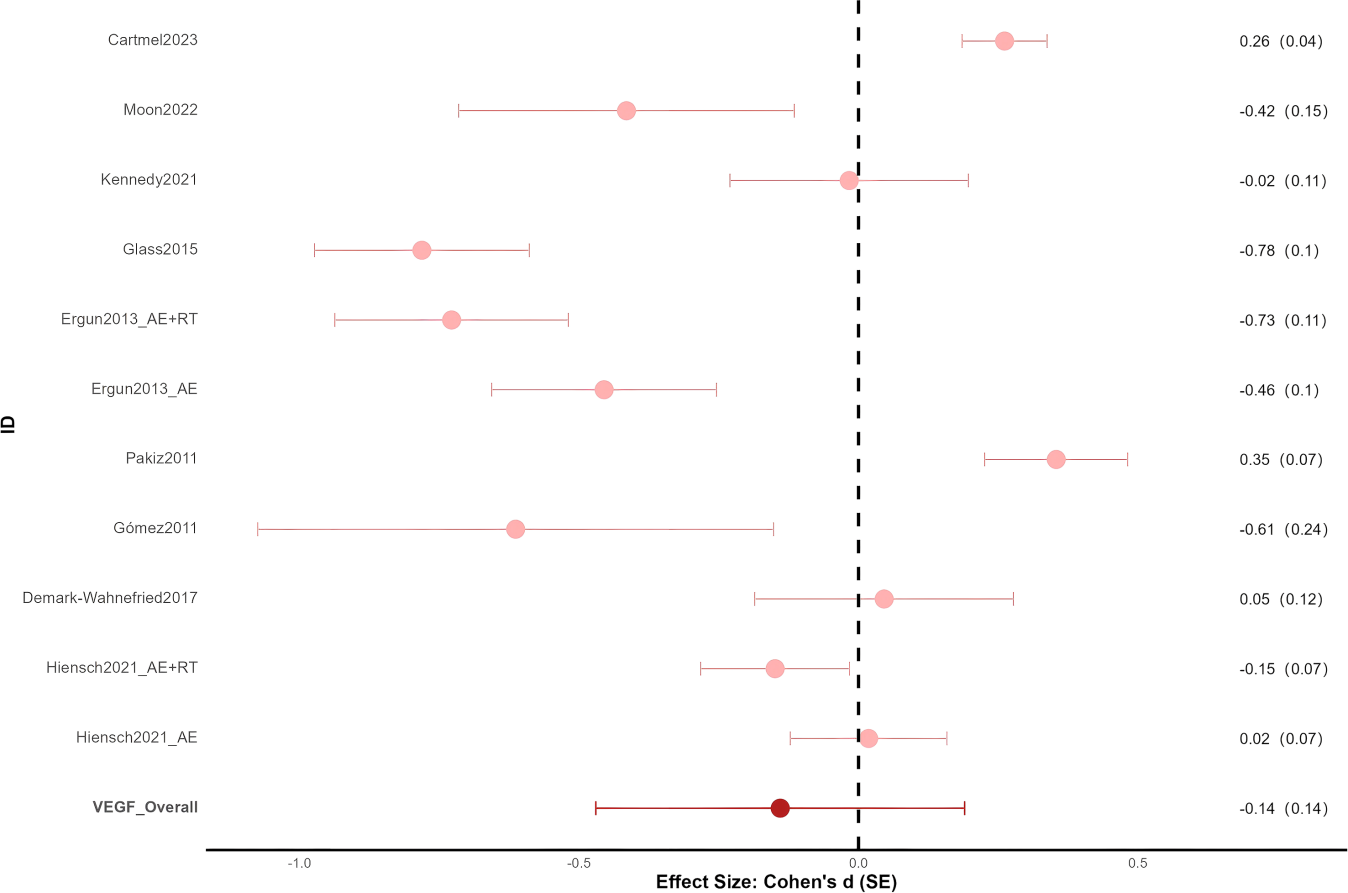
Forest plot for VEGF.

9 RCTs involving 484 cancer patients reported the effects of exercise interventions on VEGF (VEGF-A) (44–52). A total of 11 effect sizes were generated, as two multi-arm RCTs reported separate effects for combined training (aerobic exercise combined with resistance training) and aerobic exercise (AE) (48, 52). **Figure 2** shows that the overall effect of exercise interventions on VEGF was small and nonsignificant (ES = -0.14, SE = 0.14, p = 0.36, I² = 45.04%).

Exercise modalities included combined training (n = 6) and AE (n = 5). Timing of intervention was before treatment (n = 2), during treatment (n = 3), and after treatment (n = 6). Breast cancer was the most common cancer type (n = 6). One study included only overweight breast cancer patients (49). One study reported that some participants had hypertension as a comorbidity (47). Two studies did not report cancer stage (47, 48). In the remaining studies, cancer stage was not restricted to a specific category, with stages I–III being the most commonly reported.

#### 3.1.2 Effects of exercise on ICAM-1, VCAM-1, MCP-1, and VEGF-C

4 RCTs, including two multi-arm studies and a total of 172 cancer patients, reported the effects of exercise interventions on ICAM-1 and VCAM-1 (43, 46, 50, 53). 3 RCTs, including one multi-arm study and 115 patients, reported effects on MCP-1 (46, 48, 50). 3 RCTs, including one multi-arm study and 155 patients, reported effects on VEGF-C (46, 52, 54).

**Figure 3:**
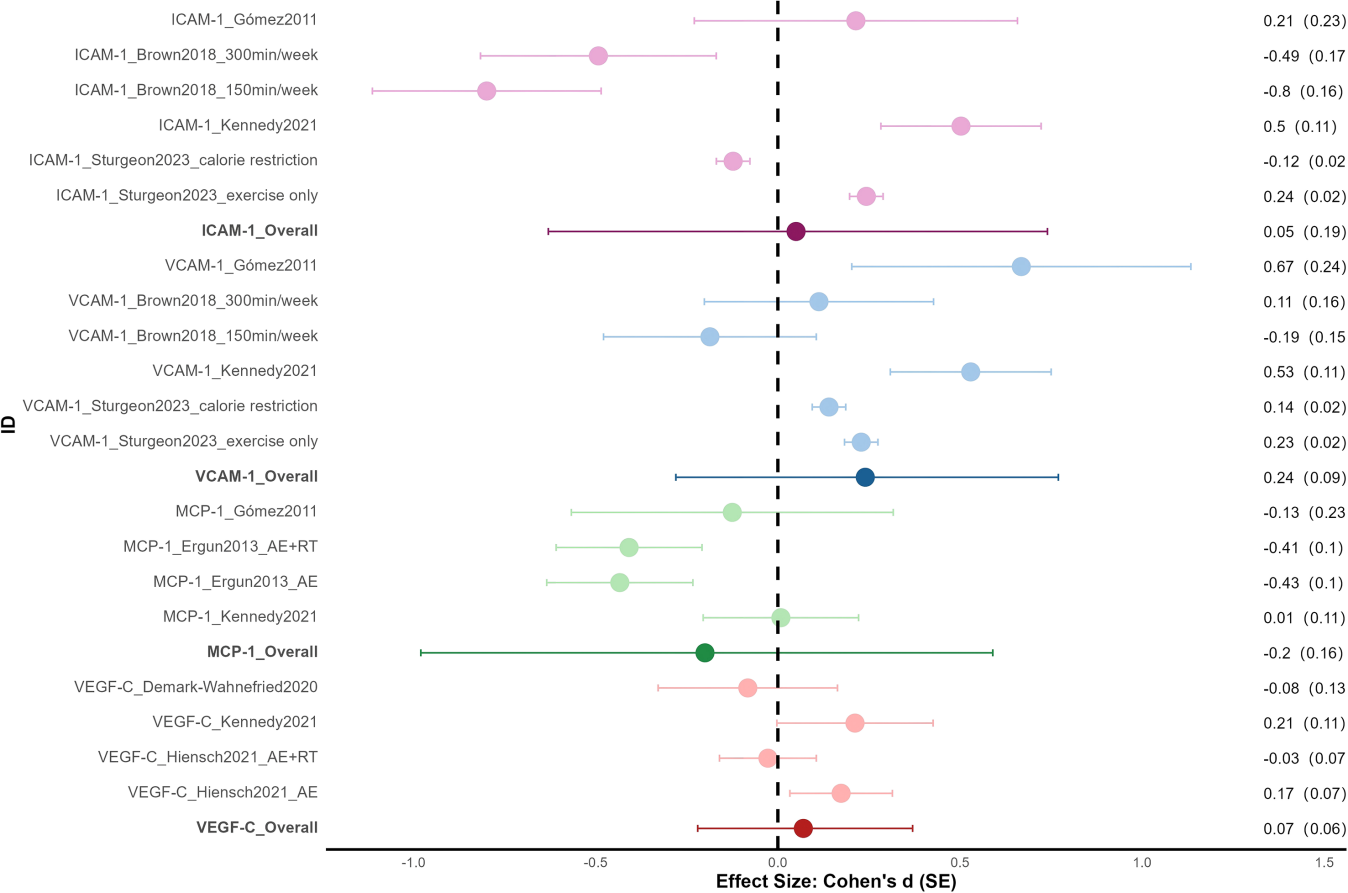
Forest plot for other angiogenesis biomarkers.

As shown in **Figure 3**, the overall effects of exercise on circulating ICAM-1, VCAM-1, and MCP-1 in cancer patients were small and nonsignificant. Specifically, VCAM-1 (ES = 0.24, SE = 0.09, p = 0.15, I² = 0%) and MCP-1 (ES = -0.20, SE = 0.16, p = 0.36, I² = 0%) reached small effect sizes but were not significant. VEGF-C (ES = 0.07, SE = 0.06, p = 0.38, I² = 0%) and ICAM-1 (ES = 0.05, SE = 0.19, p = 0.81, I² = 47.52%) showed negligible and nonsignificant pooled effect sizes.

#### 3.1.3 Other angiogenesis biomarkers

**Supplementary Material 4** (**Supplementary Figure S1**) present the effect sizes (Cohen’s d) for angiogenesis biomarkers that were reported in fewer studies. Within the VEGF family, these included VEGF-D (n = 1), PlGF (n = 3), VEGFR-1 (n = 1), and VEGFR-2 (n = 3). Other angiogenesis-related cytokines included PDGF (n = 2), PDGF subunit B (n = 2), PDGF-BB (n = 1), EGF (n = 2), HGF (n = 2), and FGF (n = 3). The ANG family included ANG (n = 2), ANG-1 (n = 2), and ANG-2 (n = 2).

### 3.2 Meta-regression results

Meta-regression results for VEGF are reported in **Supplementary Material 5**, **Table S3** details the extraction methods for 12 moderators. Parameters of the univariable RVE regression models are presented in **Supplementary Table S4**, with corresponding visualizations shown in **Supplementary Figure S2**. Among these, the supervision model explained 99% of heterogeneity (R² = 0.99, I² = 0.65%). BMI (R² = 0.49, I² = 29.48%), intervention duration (R² = 0.11, I² = 41.98%), and combination with other interventions (R² = 0.11, I² = 42.04%) also partially accounted for between-study heterogeneity. Notably, the treatment-timing model showed that exercise interventions delivered before cancer treatment (ES = -0.17) and during treatment (ES = -0.23) were more effective than those delivered after treatment (ES = 0.14).

### 3.3 Risk of bias, publication bias, and quality of evidence

Risk of bias was assessed using the RoB 2 tool. The main sources of bias included lack of preregistration, failure to report methods for handling missing data, and insufficient detail regarding exercise interventions (**Supplementary Material 6**, **Figures S3, S4**). Egger’s tests for the five outcomes yielded p values > 0.05, suggesting no significant publication bias (**Supplementary Material 6**, **Figure S5**). The certainty of evidence was assessed using the GRADE framework, with overall ratings ranging from low to very low (**Supplementary Material 6**, **Table S5**).

## 4 Discussion

This systematic review indicates that regular exercise is associated with only small and statistically nonsignificant changes in circulating angiogenesis biomarkers, including VEGF, VCAM-1, and MCP-1, in patients with cancer. Given the low certainty of evidence and the fact that these biomarkers were secondary outcomes in most included trials, these results should be interpreted cautiously. To our knowledge, this is the first systematic review and meta-analysis to examine the effects of regular exercise on angiogenesis biomarkers in this population. The following sections contextualize these preliminary findings and discuss their potential implications.

### 4.1 Effects of regular exercise on circulating VEGF in patients with cancer and its clinical implications

Our meta-analysis showed a small and statistically nonsignificant reduction in circulating VEGF among patients with cancer. Notably, this pattern differs from that observed in healthy individuals. Recent meta-analyses report that acute aerobic exercise increases circulating VEGF and VEGF mRNA levels in healthy populations (55, 56), and regular exercise can also elevate circulating VEGF in healthy older adults (57). Although elevated circulating VEGF in cancer is associated with tumor progression and poorer prognosis, it remains unclear whether the small, nonsignificant differences observed here reflect any meaningful biological response. Consequently, any hypothesis that exercise-induced VEGF changes may influence vascular function or treatment response should be regarded as preliminary and requires confirmation in rigorously designed clinical trials.

We further observed that this decrease in VEGF appeared to vary by treatment stage. Regression analyses indicated that exercise implemented before (ES = −0.17) or during cancer treatment (ES = −0.23) showed more negative VEGF estimates, whereas post-treatment survivors demonstrated a response pattern more consistent with healthy individuals (ES = 0.14). Although these differences were small and statistically nonsignificant, they suggest that exercise may exert a stronger modulatory influence on the angiogenic environment during active disease and could potentially interact with standard anticancer therapies. Future adequately powered studies are needed to determine whether treatment phase modifies the VEGF response to exercise.

Regarding exercise prescription parameters, our regression analyses revealed several noteworthy patterns. Combined aerobic and resistance training (effect size = −0.22) showed more negative VEGF estimates than aerobic exercise alone (effect size = −0.04). This aligns with findings from a network meta-analysis in cancer patients showing that combined aerobic and resistance training was superior to single-modality exercise in reducing inflammatory markers such as IL-6, IL-8, IL-10, TNF-α, and CRP (36). Moreover, two three-arm RCTs conducted during and after breast cancer treatment reported that combined aerobic and resistance training had greater effects on VEGF than aerobic exercise alone (48, 52). We also observed that higher exercise intensity may be a key driver of VEGF reduction, whereas increasing duration or total volume did not produce similar effects. This pattern is consistent with another meta-regression examining the effects of exercise on circulating IL-6 concentrations in cancer patients (58).

Although these prescription-related findings require validation in larger trials, they are broadly consistent with current exercise guidelines for cancer patients, which recommend combined aerobic and resistance training and at least 75 minutes of vigorous aerobic exercise per week (59, 60). In addition, our findings suggest that supervised training may yield more stable effects, underscoring the importance of exercise quality and adherence in practice. Importantly, none of the included RCTs reported serious exercise-related adverse events, supporting the safety of structured exercise interventions before and during cancer treatment.

Although our review did not include mind–body exercise interventions, emerging preliminary evidence suggests that such approaches may also influence angiogenesis-related biomarkers in cancer populations. For example, a randomized controlled trial in men with prostate cancer (NCT02620033) reported that a 12-week perioperative yoga program (two 60-minute sessions per week) significantly reduced MCP-1 and increased VEGF, alongside improvements in quality of life (61). Similarly, a conference abstract in breast cancer survivors described that a 16-week therapeutic yoga program was associated with post-intervention differences in VEGF-A (+45.51%) and MCP-1 (−19.79%) (62). These early findings illustrate the diversity of movement-based approaches that could interact with angiogenesis-related pathways, although their heterogeneity and limited methodological detail preclude direct comparison with structured aerobic or resistance training.

### 4.2 Mechanisms underlying the regulation of circulating VEGF by regular exercise in patients with cancer

Although direct mechanistic evidence from clinical studies is scarce, preclinical research provides several biologically plausible pathways through which exercise might influence VEGF regulation. Across various tumor models, regular exercise has been shown to downregulate VEGF expression in tumor tissues or in circulation (63–66). Further evidence indicates that regular exercise can reduce VEGF expression within breast tumor tissue, thereby decreasing angiogenesis and tumor growth (67). During chemotherapy, aerobic exercise can enhance tumor perfusion and induce vascular remodeling, improving chemotherapy efficacy (68). However, animal findings remain heterogeneous. Some studies have reported that exercise increases VEGF without altering tumor growth (69), and others have proposed that exercise-induced elevations in VEGF may help alleviate tumor burden (70). These diverse findings point to the existence of multiple interacting mechanisms.

In cancer patients, elevated VEGF primarily originates from tumor cells, tumor-associated macrophages (TAMs), and neutrophils (71, 72). Animal studies consistently show that regular exercise reduces TAM and neutrophil infiltration in tumor tissue and decreases the expression of M2 macrophage–associated markers (73–76). Although human evidence remains limited, one RCT in breast cancer survivors reported changes in adipose tissue macrophage phenotype following combined exercise training (77). These observations suggest that immune-cell pathways may contribute to VEGF modulation.

Inflammatory regulation may represent another potential pathway. Regular exercise reduces circulating pro-inflammatory cytokines such as IL-6 and TNF-α in cancer patients (36, 58). Both IL-6 and TNF-α have been shown to promote tumor angiogenesis by upregulating VEGF through signaling pathways such as STAT3 and NF-κB (78–82). Several human studies also support this inflammation–angiogenesis axis, reporting positive correlations between IL-6 or TNF-α and circulating VEGF levels in patients with breast cancer, small cell lung cancer, and melanoma (83–85). In contrast, the anti-inflammatory cytokine IL-10 suppresses VEGF production from macrophages and tumor cells and inhibits VEGF-dependent endothelial proliferation (86–88). Acute exercise induces a transient IL-6 response that promotes IL-10 secretion, while long-term training is associated with more stable anti-inflammatory adaptations (36). These observations raise the possibility that the anti-inflammatory effects of exercise may contribute to reductions in VEGF.

Adipose tissue, an important source of VEGF, may constitute another mechanism underlying exercise-induced VEGF regulation. Animal evidence shows that obesity increases circulating TNF-α and VEGF levels (89, 90). Moreover, adipose-derived VEGF strongly promotes angiogenesis and tumor growth in breast cancer and reduces the effectiveness of anti-VEGF therapies (91, 92). Exercise-induced improvements in adipose tissue quantity and quality are well established (28). An RCT in overweight women further showed that exercise-related weight loss was significantly associated with reductions in circulating VEGF, with the greatest decreases observed among those who achieved at least 10% weight loss during long-term follow-up (93, 94).

Hypoxia-inducible factors (HIFs) may represent another potential pathway. HIFs are key transcription factors mediating cellular responses to hypoxia, upregulating VEGF expression to promote angiogenesis under hypoxic conditions (95, 96). HIF-1α and HIF-2α are widely overexpressed in multiple tumor types (97, 98). Animal studies indicate that TNF-α can activate HIF through the NF-κB pathway, and HIF upregulation further drives VEGF production (96). Inhibition of HIF-1α reduces VEGF expression and suppresses tumor cell proliferation and invasion (99). Some animal models have shown that aerobic exercise decreases HIF-1α and VEGF-A expression in tumor and adipose tissues (67, 100, 101). However, almost no clinical trials have directly evaluated whether exercise modulates HIF activity in patients with cancer.

Taken together, these pathways offer biologically plausible frameworks through which exercise might influence VEGF regulation. However, given the limited mechanistic evidence in humans, these explanations should be regarded as exploratory and require targeted clinical investigation.

### 4.3 Effects of regular exercise on other angiogenesis biomarkers in patients with cancer

Although VEGF was the primary focus of this study, our findings indicate that regular exercise may also be associated with small shifts in other angiogenesis-related molecules.

We observed a slight increase in circulating vascular cell adhesion molecule-1 (VCAM-1) following regular exercise in patients with cancer. VCAM-1 is a key adhesion molecule involved in endothelial activation and angiogenesis (102). Traditionally, VCAM-1 upregulation has been thought to promote tumor angiogenesis and metastasis (103). However, emerging evidence challenges this view. Some studies have shown that VEGF may suppress VCAM-1 expression to facilitate tumor immune evasion (104). In pancreatic ductal adenocarcinoma, restoring VCAM-1 expression enhances T-cell infiltration, inhibits tumor growth, and is associated with longer survival (105). Additionally, recent animal evidence reports that aerobic exercise improves tumor vasculature and increases VCAM-1 expression in two melanoma models, accompanied by enhanced immune mobilization (106). Taken together, these findings suggest that exercise-related changes in VCAM-1 may reflect immune-activating rather than purely pro-angiogenic processes.

We also observed a reduction in circulating monocyte chemoattractant protein-1 (MCP-1) following regular exercise. MCP-1 is an important regulator within the chemotactic–inflammatory–angiogenic axis, although its value as a clinical biomarker remains inconsistent across studies (107, 108). Recent animal research indicates that MCP-1 promotes the upregulation of HIF-1α, thereby driving VEGF expression (109). In cancer patients, MCP-1 levels are positively correlated with both VEGF and TNF-α concentrations (110). Our findings align with those of a clinical study in esophageal adenocarcinoma, which reported that exercise attenuated the chemotherapy-related rise in MCP-1 (111). Given MCP-1’s upstream position in inflammatory–angiogenic signaling, its reduction could plausibly contribute to the VEGF patterns observed in our analysis.

Overall, these response patterns suggest that exercise may influence angiogenesis-related and immune-regulatory pathways beyond VEGF alone. However, these findings are preliminary, and confirmation will require rigorously designed, mechanism-oriented clinical trials.

### 4.4 Limitations and future directions

This study is not without limitations. First, due to the limited number of available RCTs, we excluded angiogenesis biomarkers originally planned for analysis, such as angiopoietins (ANG), which constrained our ability to characterize the broader angiogenesis network. Second, variability in outcome measurement and reporting of exercise prescription parameters (intensity, frequency, supervision, etc.) across studies may have affected the comparability of results. Third, given the limited number of eligible trials, random-effects estimates in both the primary analysis and RVE models may be unstable, potentially affecting the precision of the pooled effects. Fourth, part of our mechanistic interpretation was based on animal evidence, which should be regarded as exploratory and hypothesis-generating rather than confirmatory. Fifth, heterogeneity in treatment regimens and patient characteristics across cancer types may have contributed to differences in intervention effects. Sixth, in most RCTs included in this review, angiogenesis biomarkers were not reported as primary outcomes, this may have led to unstable effect estimates and limited interpretability. In addition, mind–body interventions (e.g., yoga, tai chi) were not included, which may limit the comprehensiveness of the exercise modalities evaluated. Therefore, given that the certainty of evidence based on our GRADE assessment was rated as “Low” or “Very Low,” these findings should be interpreted with caution in clinical practice.

Future research should address several directions. First, upcoming RCTs should designate angiogenesis biomarkers as primary or key secondary outcomes. Second, mediation analyses are needed to test mechanistic pathways linking inflammatory cytokines and HIF signaling to VEGF regulation. Third, studies should investigate whether the exercise-related increase in VCAM-1 reflects enhanced immune mobilization and contributes to a more favorable tumor microenvironment. Fourth, the independent and interactive effects of adipose tissue quantity and function on VEGF should be clarified. Fifth, future trials should stratify participants by cancer type, treatment stage, regimen, and metabolic phenotype (e.g., BMI) to identify treatable subgroups. Sixth, dose–response relationships of exercise intensity on angiogenesis biomarkers warrant investigation. Seventh, integrating exercise with conventional therapies may help evaluate the translational potential of exercise-induced vascular normalization. Additionally, harmonizing biomarker assay procedures would improve comparability across studies. Future work should also assess mind–body exercise modalities within the same analytical framework.

## 5 Conclusion

Regular exercise was associated with only small and nonsignificant changes in angiogenesis biomarkers in patients with cancer. These preliminary patterns suggest possible interactions with angiogenic and immune pathways, but the low certainty of evidence limits firm conclusions. Future randomized controlled trials are needed to confirm these changes and examine how exercise prescriptions at different treatment stages influence these biomarkers.

## Data Availability

The original contributions presented in the study are included in the article/**Supplementary Material**. Further inquiries can be directed to the corresponding author.

## References

[1] Cao Y Langer R Ferrara N . Targeting angiogenesis in oncology, ophthalmology and beyond. Nat Rev Drug Discov. (2023) 22:476–95. doi: 10.1038/s41573-023-00671-z, PMID: 37041221

[2] Lugano R Ramachandran M Dimberg A . Tumor angiogenesis: causes, consequences, challenges and opportunities. Cell Mol Life Sci. (2020) 77:1745–70. doi: 10.1007/s00018-019-03351-7, PMID: 31690961 PMC7190605

[3] Liu ZL Chen HH Zheng LL Sun LP Shi L . Angiogenic signaling pathways and anti-angiogenic therapy for cancer. Signal Transduct Target Ther. (2023) 8:198. doi: 10.1038/s41392-023-01460-1, PMID: 37169756 PMC10175505

[4] Martin JD Seano G Jain RK . Normalizing function of tumor vessels: progress, opportunities, and challenges. Annu Rev Physiol. (2019) 81:505–34. doi: 10.1146/annurev-physiol-020518-114700, PMID: 30742782 PMC6571025

[5] Patel SA Nilsson MB Le X Cascone T Jain RK Heymach JV . Molecular mechanisms and future implications of VEGF/VEGFR in cancer therapy. Clin Cancer Res. (2023) 29:30–9. doi: 10.1158/1078-0432.CCR-22-1366, PMID: 35969170 PMC10274152

[6] Carmeliet P . VEGF as a key mediator of angiogenesis in cancer. Oncology. (2005) 69 Suppl 3:4–10. doi: 10.1159/000088478, PMID: 16301830

[7] Yang Y Cao Y . The impact of VEGF on cancer metastasis and systemic disease. Semin Cancer Biol. (2022) 86:251–61. doi: 10.1016/j.semcancer.2022.03.011, PMID: 35307547

[8] Wu H Ma T Li D He M Wang H Cui Y . Circulating vascular endothelial growth factor and cancer risk: A bidirectional mendelian randomization. Front Genet. (2022) 13:981032. doi: 10.3389/fgene.2022.981032, PMID: 36159967 PMC9489904

[9] Liang B He Q Zhong L Wang S Pan Z Wang T . Circulating VEGF as a biomarker for diagnosis of ovarian cancer: a systematic review and a meta-analysis. Onco Targets Ther. (2015) 8:1075–82. doi: 10.2147/OTT.S83616, PMID: 26028975 PMC4440429

[10] Cai C Wang X Fu Q Chen A . The VEGF expression associated with prognosis in patients with intrahepatic cholangiocarcinoma: a systematic review and meta-analysis. World J Surg Oncol. (2022) 20:40. doi: 10.1186/s12957-022-02511-7, PMID: 35189920 PMC8859901

[11] Hu P Liu W Wang L Yang M Du J . High circulating VEGF level predicts poor overall survival in lung cancer. J Cancer Res Clin Oncol. (2013) 139:1157–67. doi: 10.1007/s00432-013-1425-1, PMID: 23552871 PMC11824210

[12] Fahmi MN Pradjatmo H Astuti I Nindrea RD . Cytokines as prognostic biomarkers of epithelial ovarian cancer (EOC): A systematic review and meta-analysis. Asian Pac J Cancer Prev. (2021) 22:315–23. doi: 10.31557/APJCP.2021.22.2.315, PMID: 33639643 PMC8190346

[13] Banys-Paluchowski M Witzel I Riethdorf S Pantel K Rack B Janni W . The clinical relevance of serum vascular endothelial growth factor (VEGF) in correlation to circulating tumor cells and other serum biomarkers in patients with metastatic breast cancer. Breast Cancer Res Treat. (2018) 172:93–104. doi: 10.1007/s10549-018-4882-z, PMID: 30003393

[14] Zhan P Ji YN Yu LK . VEGF is associated with the poor survival of patients with prostate cancer: a meta-analysis. Transl Androl Urol. (2013) 2:99–105. doi: 10.3978/j.issn.2223-4683.2013.06.03, PMID: 26816732 PMC4708223

[15] Des Guetz G Uzzan B Nicolas P Cucherat M Morere JF Benamouzig R . Microvessel density and VEGF expression are prognostic factors in colorectal cancer. Meta-analysis literature. Br J Cancer. (2006) 94:1823–32. doi: 10.1038/sj.bjc.6603176, PMID: 16773076 PMC2361355

[16] Tanaka K Sugisaka J Shiraishi Y Watanabe Y Daga H Azuma K . Serum VEGF-A as a biomarker for the addition of bevacizumab to chemo-immunotherapy in metastatic NSCLC. Nat Commun. (2025) 16:2825. doi: 10.1038/s41467-025-58186-7, PMID: 40121197 PMC11929838

[17] Santos LV Cruz MR Lopes Gde L Lima JP . VEGF-A levels in bevacizumab-treated breast cancer patients: a systematic review and meta-analysis. Breast Cancer Res Treat. (2015) 151:481–9. doi: 10.1007/s10549-015-3410-7, PMID: 25947646

[18] Conway JW Braden J Lo SN Scolyer RA Carlino MS Menzies AM . VEGF Inhibitors Improve Survival Outcomes Patients Liver Metastases across Cancer Types-A Meta-Analysis. Cancers. (2023) 15:5012. doi: 10.3390/cancers15205012, PMID: 37894379 PMC10605052

[19] Huang D Ke L Cui H Li S Sun F . Efficacy and safety of VEGF/VEGFR inhibitors for platinum-resistant ovarian cancer: a systematic review and meta-analysis of randomized controlled trials. BMC Womens Health. (2024) 24:34. doi: 10.1186/s12905-023-02879-y, PMID: 38218775 PMC10788010

[20] Wagner AD Thomssen C Haerting J Unverzagt S . Vascular-endothelial-growth-factor (VEGF) targeting therapies for endocrine refractory or resistant metastatic breast cancer. Cochrane Database Syst Rev. (2012) 2012:Cd008941. doi: 10.1002/14651858.CD008941.pub2, PMID: 22786517 PMC12066189

[21] He H Zhou F . Efficacy and safety of anti-angiogenic drugs combined with chemotherapy in the treatment of platinum-sensitive/resistant ovarian cancer: a meta-analysis with trial sequential analysis of randomized controlled trials. Front Pharmacol. (2024) 15:1446403. doi: 10.3389/fphar.2024.1446403, PMID: 39640492 PMC11617189

[22] Michalak S Kalinowska-Lyszczarz A Rybacka-Mossakowska J Zaborowski M Kozubski W . The associations between serum vascular endothelial growth factor, tumor necrosis factor and interleukin 4 with the markers of blood-brain barrier breakdown in patients with paraneoplastic neurological syndromes. J Neural Transm (Vienna). (2019) 126:149–58. doi: 10.1007/s00702-018-1950-9, PMID: 30374596 PMC6373237

[23] Ding GX Feng CC Song NH Fang ZJ Xia GW Jiang HW . Paraneoplastic symptoms: cachexia, polycythemia, and hypercalcemia are, respectively, related to vascular endothelial growth factor (VEGF) expression in renal clear cell carcinoma. Urol Oncol. (2013) 31:1820–5. doi: 10.1016/j.urolonc.2012.03.021, PMID: 22534085

[24] de Jong MA Slingerland M Hawinkels L Nielsen M Crobach A de Jonge-Muller ESM . Recurrent paraneoplastic nephrotic syndrome; insights from a Lynch syndrome patient with multiple Malignancies. Fam Cancer. (2024) 24:11. doi: 10.1007/s10689-024-00435-7, PMID: 39630202

[25] Zhu C Ma H He A Li Y He C Xia Y . Exercise in cancer prevention and anticancer therapy: Efficacy, molecular mechanisms and clinical information. Cancer Lett. (2022) 544:215814. doi: 10.1016/j.canlet.2022.215814, PMID: 35803475

[26] Koelwyn GJ Quail DF Zhang X White RM Jones LW . Exercise-dependent regulation of the tumour microenvironment. Nat Rev Cancer. (2017) 17:620–32. doi: 10.1038/nrc.2017.78, PMID: 28943640

[27] Koelwyn GJ Zhuang X Tammela T Schietinger A Jones LW . Exercise and immunometabolic regulation in cancer. Nat Metab. (2020) 2:849–57. doi: 10.1038/s42255-020-00277-4, PMID: 32929232 PMC9128397

[28] He A Pu Y Jia C Wu M He H Xia Y . The influence of exercise on cancer risk, the tumor microenvironment and the treatment of cancer. Sports Med. (2024) 54:1371–97. doi: 10.1007/s40279-024-02031-2, PMID: 38687441

[29] Esteves M Monteiro MP Duarte JA . The effects of physical exercise on tumor vasculature: systematic review and meta-analysis. Int J Sports Med. (2021) 42:1237–49. doi: 10.1055/a-1533-1876, PMID: 34341974

[30] Seet-Lee C Yee J Morahan H Ross LS Edwards KM . The effect of aerobic exercise on tumour blood delivery: a systematic review and meta-analysis. Support Care Cancer. (2022) 30:8637–53. doi: 10.1007/s00520-022-07132-0, PMID: 35650456 PMC9633495

[31] Beaudry RI Liang Y Boyton ST Tucker WJ Brothers RM Daniel KM . Meta-analysis of exercise training on vascular endothelial function in cancer survivors. Integr Cancer Ther. (2018) 17:192–9. doi: 10.1177/1534735418756193, PMID: 29390904 PMC6041934

[32] Hu C Tang J Gao Y Cao R . Effects of physical exercise on body fat and laboratory biomarkers in cancer patients: a meta-analysis of 35 randomized controlled trials. Support Care Cancer. (2022) 30:1–12. doi: 10.1007/s00520-022-07013-6, PMID: 35501513

[33] Tipton E . Small sample adjustments for robust variance estimation with meta-regression. Psychol Methods. (2015) 20:375–93. doi: 10.1037/met0000011, PMID: 24773356

[34] Hedges LV Tipton E Johnson MC . Robust variance estimation in meta-regression with dependent effect size estimates. Res Synth Methods. (2010) 1:39–65. doi: 10.1002/jrsm.5, PMID: 26056092

[35] Page MJ McKenzie JE Bossuyt PM Boutron I Hoffmann TC Mulrow CD . The PRISMA 2020 statement: an updated guideline for reporting systematic reviews. Bmj. (2021) 372:n71. doi: 10.1136/bmj.n71, PMID: 33782057 PMC8005924

[36] Wang J He Y Kim AR Lee KH Choi SW . Effects of different types of exercise on inflammatory markers in cancer patients: A systematic review and Bayesian network meta-analysis. J Sports Sci. (2025) 43:1121–38. doi: 10.1080/02640414.2025.2486886, PMID: 40197224

[37] Cumpston M Li T Page MJ Chandler J Welch VA Higgins JP . Updated guidance for trusted systematic reviews: a new edition of the Cochrane Handbook for Systematic Reviews of Interventions. Cochrane Database Syst Rev. (2019) 10:Ed000142. doi: 10.1002/14651858.ED000142, PMID: 31643080 PMC10284251

[38] Sterne JAC Savović J Page MJ Elbers RG Blencowe NS Boutron I . RoB 2: a revised tool for assessing risk of bias in randomised trials. Bmj. (2019) 366:l4898. doi: 10.1136/bmj.l4898, PMID: 31462531

[39] Guyatt G Oxman AD Akl EA Kunz R Vist G Brozek J . GRADE guidelines: 1. Introduction-GRADE evidence profiles and summary of findings tab les. J Clin Epidemiol. (2011) 64:383–94. doi: 10.1016/j.jclinepi.2010.04.026, PMID: 21195583

[40] Nakagawa S Cuthill IC . Effect size, confidence interval and statistical significance: a practical guide for biologists. Biol Rev Camb Philos Soc. (2007) 82:591–605. doi: 10.1111/j.1469-185X.2007.00027.x, PMID: 17944619

[41] Huedo-Medina TB Sánchez-Meca J Marín-Martínez F Botella J . Assessing heterogeneity in meta-analysis: Q statistic or I2 index? Psychol Methods. (2006) 11:193–206. doi: 10.1037/1082-989X.11.2.193, PMID: 16784338

[42] Plonsky L Ghanbar H . Multiple regression in L2 research: A methodological synthesis and guide to interpreting R2 values. Modern Lang J. (2018) 102:713–31. doi: 10.1111/modl.12509

[43] Brown JC Troxel AB Ky B Damjanov N Zemel BS Rickels MR . Dose-response effects of aerobic exercise among colon cancer survivors: A randomized phase II trial. Clin Colorectal Cancer. (2018) 17:32–40. doi: 10.1016/j.clcc.2017.06.001, PMID: 28669606 PMC5733696

[44] Cartmel B Li FY Zhou Y Gottlieb L Lu L Mszar R . Randomized trial of exercise on cancer-related blood biomarkers and survival in women with ovarian cancer. Cancer Med. (2023) 12:15492–503. doi: 10.1002/cam4.6187, PMID: 37269192 PMC10417064

[45] Moon C Gallegos AM Sheikh B Kumar P Liss M Patel DI . Pilot study on the impact of a home-based exercise program on inflammatory cytokines and quality of life in men with prostate cancer under active surveillance. Cancer Control. (2022) 29:10732748221130964. doi: 10.1177/10732748221130964, PMID: 36200522 PMC9549098

[46] Kennedy SA Annett SL Dunne MR Boland F O’Neill LM Guinan EM . Effect of the rehabilitation program, reStOre, on serum biomarkers in a randomized control trial of esophagogastric cancer survivors. Front Oncol. (2021) 11:669078. doi: 10.3389/fonc.2021.669078, PMID: 34604026 PMC8479183

[47] Glass OK Inman BA Broadwater G Courneya KS Mackey JR Goruk S . Effect of aerobic training on the host systemic milieu in patients with solid tumours: an exploratory correlative study. Br J Cancer. (2015) 112:825–31. doi: 10.1038/bjc.2014.662, PMID: 25584487 PMC4453949

[48] Ergun M Eyigor S Karaca B Kisim A Uslu R . Effects of exercise on angiogenesis and apoptosis-related molecules, quality of life, fatigue and depression in breast cancer patients. Eur J Cancer Care (Engl). (2013) 22:626–37. doi: 10.1111/ecc.12068, PMID: 23731173

[49] Pakiz B Flatt SW Bardwell WA Rock CL Mills PJ . Effects of a weight loss intervention on body mass, fitness, and inflammatory biomarkers in overweight or obese breast cancer survivors. Int J Behav Med. (2011) 18:333–41. doi: 10.1007/s12529-010-9079-8, PMID: 21336679 PMC3212681

[50] Gómez AM Martínez C Fiuza-Luces C Herrero F Pérez M Madero L . Exercise training and cytokines in breast cancer survivors. Int J Sports Med. (2011) 32:461–7. doi: 10.1055/s-0031-1271697, PMID: 21380980

[51] Demark-Wahnefried W Rais-Bahrami S Desmond RA Gordetsky JB Hunter GR Yang ES . Presurgical weight loss affects tumour traits and circulating biomarkers in men with prostate cancer. Br J Cancer. (2017) 117:1303–13. doi: 10.1038/bjc.2017.303, PMID: 28881355 PMC5672928

[52] Hiensch AE Mijwel S Bargiela D Wengström Y May AM Rundqvist H . Inflammation mediates exercise effects on fatigue in patients with breast cancer. Med Sci Sports Exerc. (2021) 53:496–504. doi: 10.1249/MSS.0000000000002490, PMID: 32910094 PMC7886356

[53] Sturgeon KM Brown JC Sears DD Sarwer DB Schmitz KH . WISER survivor trial: combined effect of exercise and weight loss interventions on inflammation in breast cancer survivors. Med Sci Sports Exerc. (2023) 55:209–15. doi: 10.1249/MSS.0000000000003050, PMID: 36170550 PMC9840668

[54] Demark-Wahnefried W Rogers LQ Gibson JT Harada S Frugé AD Oster RA . Randomized trial of weight loss in primary breast cancer: Impact on body composition, circulating biomarkers and tumor characteristics. Int J Cancer. (2020) 146:2784–96. doi: 10.1002/ijc.32637, PMID: 31442303 PMC7155016

[55] Song BX Azhar L Koo GKY Marzolini S Gallagher D Swardfager W . The effect of exercise on blood concentrations of angiogenesis markers in older adults: A systematic review and meta-analysis. Neurobiol Aging. (2024) 135:15–25. doi: 10.1016/j.neurobiolaging.2023.12.004, PMID: 38147807

[56] Aragón-Vela J Casuso RA . Effect of hypoxia-inducible factor 1 on vascular endothelial growth factor expression in exercised human skeletal muscle: a systematic review and meta-analysis. Am J Physiol Cell Physiol. (2025) 329:C272–c82. doi: 10.1152/ajpcell.00297.2025, PMID: 40522862

[57] Behrad S Dezfuli SAT Yazdani R Hayati S Shanjani SM . The effect of physical exercise on circulating neurotrophic factors in healthy aged subjects: A meta-analysis and meta-regression. Exp Gerontol. (2024) 196:112579. doi: 10.1016/j.exger.2024.112579, PMID: 39260585

[58] Wang J He Y Wang Z Wang Z Miao Y Choi J-Y . Effects of different exercise prescription parameters on metabolic and inflammatory biomarkers in cancer patients: A systematic review, meta-analysis, and meta-regression. Front Immunol. (2025) 16:1663560. doi: 10.3389/fimmu.2025.1663560, PMID: 40895542 PMC12390815

[59] Buffart LM Galvão DA Brug J Chinapaw MJ Newton RU . Evidence-based physical activity guidelines for cancer survivors: current guidelines, knowledge gaps and future research directions. Cancer Treat Rev. (2014) 40:327–40. doi: 10.1016/j.ctrv.2013.06.007, PMID: 23871124

[60] Rock CL Thomson C Gansler T Gapstur SM McCullough ML Patel AV . American Cancer Society guideline for diet and physical activity for cancer prevention. CA Cancer J Clin. (2020) 70:245–71. doi: 10.3322/caac.21591, PMID: 32515498

[61] Kaushik D Shah PK Mukherjee N Ji N Dursun F Kumar AP . Effects of yoga in men with prostate cancer on quality of life and immune response: a pilot randomized controlled trial. Prostate Cancer Prostatic Dis. (2022) 25:531–8. doi: 10.1038/s41391-021-00470-w, PMID: 34815548 PMC9124736

[62] Sonawane M Almeida G Darby N Serra M Calderon T Lapetoda A . Abstract P3-01-28: therapeutic yoga enhances cognitive and metabolic markers in breast cancer survivors. Clin Cancer Res. (2025) 31:P3–01-28-P3-01-28. doi: 10.1158/1557-3265.SABCS24-P3-01-28

[63] Shirali S Barari A Hosseini SA Khodadi E . Effects of six weeks endurance training and aloe vera supplementation on COX-2 and VEGF levels in mice with breast cancer. Asian Pac J Cancer Prev. (2017) 18:31–6. doi: 10.22034/APJCP.2017.18.1.31, PMID: 28240006 PMC5563116

[64] Shalamzari SA Agha-Alinejad H Alizadeh S Shahbazi S Khatib ZK Kazemi A . The effect of exercise training on the level of tissue IL-6 and vascular endothelial growth factor in breast cancer bearing mice. Iran J Basic Med Sci. (2014) 17:231–58., PMID: 24904714 PMC4046231

[65] Rafiei MM Soltani R Kordi MR Nouri R Gaeini AA . Gene expression of angiogenesis and apoptotic factors in female BALB/c mice with breast cancer after eight weeks of aerobic training. Iran J Basic Med Sci. (2021) 24:1196–202. doi: 10.22038/ijbms.2021.55582.12427, PMID: 35083006 PMC8751744

[66] Morrisson MJ Bi F Yang K Cady SL Hartwich TM Cerchia AP . Effect of exercise on peritoneal microenvironment and progression of ovarian cancer. Am J Cancer Res. (2021) 11:5045–62., PMID: 34765311 PMC8569339

[67] Isanejad A Alizadeh AM Amani Shalamzari S Khodayari H Khodayari S Khori V . MicroRNA-206, let-7a and microRNA-21 pathways involved in the anti-angiogenesis effects of the interval exercise training and hormone therapy in breast cancer. Life Sci. (2016) 151:30–40. doi: 10.1016/j.lfs.2016.02.090, PMID: 26924493

[68] SChadler KL Thomas NJ Galie PA Bhang DH Roby KC Addai P . Tumor vessel normalization after aerobic exercise enhances chemotherapeutic efficacy. Oncotarget. (2016) 7:65429–40. doi: 10.18632/oncotarget.11748, PMID: 27589843 PMC5323166

[69] Tsai MS Kuo ML Chang CC Wu YT . The effects of exercise training on levels of vascular endothelial growth factor in tumor-bearing mice. Cancer biomark. (2013) 13:307–13. doi: 10.3233/CBM-130359, PMID: 24440969 PMC12928301

[70] Faustino-Rocha AI Silva A Gabriel J Gil da Costa RM Moutinho M Oliveira PA . Long-term exercise training as a modulator of mammary cancer vascularization. BioMed Pharmacother. (2016) 81:273–80. doi: 10.1016/j.biopha.2016.04.030, PMID: 27261604

[71] Valković T Dobrila F Melato M Sasso F Rizzardi C Jonjić N . Correlation between vascular endothelial growth factor, angiogenesis, and tumor-associated macrophages in invasive ductal breast carcinoma. Virchows Arch. (2002) 440:583–8. doi: 10.1007/s004280100458, PMID: 12070596

[72] Kusumanto YH Dam WA Hospers GA Meijer C Mulder NH . Platelets and granulocytes, in particular the neutrophils, form important compartments for circulating vascular endothelial growth factor. Angiogenesis. (2003) 6:283–7. doi: 10.1023/B:AGEN.0000029415.62384.ba, PMID: 15166496

[73] Zielinski MR Muenchow M Wallig MA Horn PL Woods JA . Exercise delays allogeneic tumor growth and reduces intratumoral inflammation and vascularization. J Appl Physiol (1985). (2004) 96:2249–56. doi: 10.1152/japplphysiol.01210.2003, PMID: 15020578 PMC3645346

[74] Almeida PW Gomes-Filho A Ferreira AJ Rodrigues CE Dias-Peixoto MF Russo RC . Swim training suppresses tumor growth in mice. J Appl Physiol (1985). (2009) 107:261–5. doi: 10.1152/japplphysiol.00249.2009, PMID: 19478194

[75] Kim MK Kim Y Park S Kim E Kim Y Kim Y . Effects of steady low-intensity exercise on high-fat diet stimulated breast cancer progression via the alteration of macrophage polarization. Integr Cancer Ther. (2020) 19:1534735420949678. doi: 10.1177/1534735420949678, PMID: 32909498 PMC7493231

[76] McClellan JL Steiner JL Day SD Enos RT Davis MJ Singh UP . Exercise effects on polyp burden and immune markers in the ApcMin/+ mouse model of intestinal tumorigenesis. Int J Oncol. (2014) 45:861–8. doi: 10.3892/ijo.2014.2457, PMID: 24859893 PMC4432723

[77] Dieli-Conwright CM Parmentier JH Sami N Lee K Spicer D Mack WJ . Adipose tissue inflammation in breast cancer survivors: effects of a 16-week combined aerobic and resistance exercise training intervention. Breast Cancer Res Treat. (2018) 168:147–57. doi: 10.1007/s10549-017-4576-y, PMID: 29168064 PMC6233989

[78] Wei LH Kuo ML Chen CA Chou CH Lai KB Lee CN . Interleukin-6 promotes cervical tumor growth by VEGF-dependent angiogenesis via a STAT3 pathway. Oncogene. (2003) 22:1517–27. doi: 10.1038/sj.onc.1206226, PMID: 12629515

[79] Kulbe H Thompson R Wilson JL Robinson S Hagemann T Fatah R . The inflammatory cytokine tumor necrosis factor-alpha generates an autocrine tumor-promoting network in epithelial ovarian cancer cells. Cancer Res. (2007) 67:585–92. doi: 10.1158/0008-5472.CAN-06-2941, PMID: 17234767 PMC2679985

[80] Yoshida S Ono M Shono T Izumi H Ishibashi T Suzuki H . Involvement of interleukin-8, vascular endothelial growth factor, and basic fibroblast growth factor in tumor necrosis factor alpha-dependent angiogenesis. Mol Cell Biol. (1997) 17:4015–23. doi: 10.1128/MCB.17.7.4015, PMID: 9199336 PMC232254

[81] Ma X Yao H Yang Y Jin L Wang Y Wu L . miR-195 suppresses abdominal aortic aneurysm through the TNF-α/NF-κB and VEGF/PI3K/Akt pathway. Int J Mol Med. (2018) 41:2350–8. doi: 10.3892/ijmm.2018.3426, PMID: 29393364

[82] Xiao Z Liu Q Mao F Wu J Lei T . TNF-α-induced VEGF and MMP-9 expression promotes hemorrhagic transformation in pituitary adenomas. Int J Mol Sci. (2011) 12:4165–79. doi: 10.3390/ijms12064165, PMID: 21747731 PMC3131615

[83] Benoy I Salgado R Colpaert C Weytjens R Vermeulen PB Dirix LY . Serum interleukin 6, plasma VEGF, serum VEGF, and VEGF platelet load in breast cancer patients. Clin Breast Cancer. (2002) 2:311–5. doi: 10.3816/CBC.2002.n.008, PMID: 11899364

[84] Wójcik E Jakubowicz J Skotnicki P Sas-Korczyńska B Kulpa JK . IL-6 and VEGF in small cell lung cancer patients. Anticancer Res. (2010) 30:1773–8. 20592377

[85] Wang X Montoyo-Pujol YG Bermudez S Corpas G Martin A Almazan F . Serum cytokine profiles of melanoma patients and their association with tumor progression and metastasis. J Oncol. (2021) 2021:6610769. doi: 10.1155/2021/6610769, PMID: 33574842 PMC7861916

[86] Wu WK Llewellyn OP Bates DO Nicholson LB Dick AD . IL-10 regulation of macrophage VEGF production is dependent on macrophage polarisation and hypoxia. Immunobiology. (2010) 215:796–803. doi: 10.1016/j.imbio.2010.05.025, PMID: 20692534

[87] Kohno T Mizukami H Suzuki M Saga Y Takei Y Shimpo M . Interleukin-10-mediated inhibition of angiogenesis and tumor growth in mice bearing VEGF-producing ovarian cancer. Cancer Res. (2003) 63:5091–4., PMID: 12941839

[88] Cervenak L Morbidelli L Donati D Donnini S Kambayashi T Wilson JL . Abolished angiogenicity and tumorigenicity of Burkitt lymphoma by interleukin-10. Blood. (2000) 96:2568–73., PMID: 11001913

[89] Aboudeya HM Abdou AS Attia MM Shaker SA Younis SA . Possible role of moderate exercise training in modulating gene expression of adipose tissue remodeling markers in obese male rats. Sport Sci Health. (2024) 20:1291–304. doi: 10.1007/s11332-024-01206-8

[90] Chen CT Du Y Yamaguchi H Hsu JM Kuo HP Hortobagyi GN . Targeting the IKKβ/mTOR/VEGF signaling pathway as a potential therapeutic strategy for obesity-related breast cancer. Mol Cancer Ther. (2012) 11:2212–21. doi: 10.1158/1535-7163.MCT-12-0180, PMID: 22826466 PMC3712288

[91] Gu JW Young E Patterson SG Makey KL Wells J Huang M . Postmenopausal obesity promotes tumor angiogenesis and breast cancer progression in mice. Cancer Biol Ther. (2011) 11:910–7. doi: 10.4161/cbt.11.10.15473, PMID: 21451264 PMC3230297

[92] Incio J Ligibel JA McManus DT Suboj P Jung K Kawaguchi K . Obesity promotes resistance to anti-VEGF therapy in breast cancer by up-regulating IL-6 and potentially FGF-2. Sci Transl Med. (2018) 10:eaag0945. doi: 10.1126/scitranslmed.aag0945, PMID: 29540614 PMC5936748

[93] Duggan C Tapsoba Jde D Wang CY McTiernan A . Dietary weight loss and exercise effects on serum biomarkers of angiogenesis in overweight postmenopausal women: A randomized controlled trial. Cancer Res. (2016) 76:4226–35. doi: 10.1158/0008-5472.CAN-16-0399, PMID: 27417562 PMC5033683

[94] Duggan C Tapsoba JD Wang CY Schubert KEF McTiernan A . Long-term effects of weight loss and exercise on biomarkers associated with angiogenesis. Cancer Epidemiol Biomarkers Prev. (2017) 26:1788–94. doi: 10.1158/1055-9965.EPI-17-0356, PMID: 29042415 PMC5712238

[95] Zhang J Yao M Xia S Zeng F Liu Q . Systematic and comprehensive insights into HIF-1 stabilization under normoxic conditions: implications for cellular adaptation and therapeutic strategies in cancer. Cell Mol Biol Lett. (2025) 30:2. doi: 10.1186/s11658-024-00682-7, PMID: 39757165 PMC11702238

[96] Jin F Zheng X Yang Y Yao G Ye L Doeppner TR . Impairment of hypoxia-induced angiogenesis by LDL involves a HIF-centered signaling network linking inflammatory TNFα and angiogenic VEGF. Aging (Albany NY). (2019) 11:328–49. doi: 10.18632/aging.101726, PMID: 30659163 PMC6366960

[97] Rashid M Zadeh LR Baradaran B Molavi O Ghesmati Z Sabzichi M . Up-down regulation of HIF-1α in cancer progression. Gene. (2021) 798:145796. doi: 10.1016/j.gene.2021.145796, PMID: 34175393

[98] Befani C Liakos P . The role of hypoxia-inducible factor-2 alpha in angiogenesis. J Cell Physiol. (2018) 233:9087–98. doi: 10.1002/jcp.26805, PMID: 29968905

[99] Zhang J Xu J Dong Y Huang B . Down-regulation of HIF-1α inhibits the proliferation, migration, and invasion of gastric cancer by inhibiting PI3K/AKT pathway and VEGF expression. Biosci Rep. (2018) 38:BSR20180741. doi: 10.1042/BSR20180741, PMID: 29899167 PMC6435555

[100] Li J Liu L Cheng Y Xie Q Wu M Chen X . Swimming attenuates tumor growth in CT-26 tumor-bearing mice and suppresses angiogenesis by mediating the HIF-1α/VEGFA pathway. Open Life Sci. (2022) 17:121–30. doi: 10.1515/biol-2022-0009, PMID: 35291563 PMC8886589

[101] Ahmadi-Kani Golzar F Fathi R Mahjoub S . High-fat diet leads to adiposity and adipose tissue inflammation: the effect of whey protein supplementation and aerobic exercise training. Appl Physiol Nutr Metab. (2019) 44:255–62. doi: 10.1139/apnm-2018-0307, PMID: 30107135

[102] Kong DH Kim YK Kim MR Jang JH Lee S . Emerging roles of vascular cell adhesion molecule-1 (VCAM-1) in immunological disorders and cancer. Int J Mol Sci. (2018) 19:1057. doi: 10.3390/ijms19041057, PMID: 29614819 PMC5979609

[103] Schlesinger M Bendas G . Vascular cell adhesion molecule-1 (VCAM-1)–an increasing insight into its role in tumorigenicity and metastasis. Int J Cancer. (2015) 136:2504–14. doi: 10.1002/ijc.28927, PMID: 24771582

[104] Huijbers EJM Khan KA Kerbel RS Griffioen AW . Tumors resurrect an embryonic vascular program to escape immunity. Sci Immunol. (2022) 7:eabm6388. doi: 10.1126/sciimmunol.abm6388, PMID: 35030032

[105] Nakajima K Ino Y Yamazaki-Itoh R Naito C Shimasaki M Takahashi M . IAP inhibitor, Embelin increases VCAM-1 levels on the endothelium, producing lymphocytic infiltration and antitumor immunity. Oncoimmunology. (2020) 9:1838812. doi: 10.1080/2162402X.2020.1838812, PMID: 33178497 PMC7595596

[106] Savage H Pareek S Lee J Ballarò R Conterno Minussi D Hayek K . Aerobic exercise alters the melanoma microenvironment and modulates ERK5 S496 phosphorylation. Cancer Immunol Res. (2023) 11:1168–83. doi: 10.1158/2326-6066.CIR-22-0465, PMID: 37307577 PMC10527747

[107] Wang L Lan J Tang J Luo N . MCP-1 targeting: Shutting off an engine for tumor development. Oncol Lett. (2022) 23:26. doi: 10.3892/ol.2021.13144, PMID: 34868363 PMC8630816

[108] Yoshimura T Li C Wang Y Matsukawa A . The chemokine monocyte chemoattractant protein-1/CCL2 is a promoter of breast cancer metastasis. Cell Mol Immunol. (2023) 20:714–38. doi: 10.1038/s41423-023-01013-0, PMID: 37208442 PMC10310763

[109] Sun C Li X Guo E Li N Zhou B Lu H . MCP-1/CCR-2 axis in adipocytes and cancer cell respectively facilitates ovarian cancer peritoneal metastasis. Oncogene. (2020) 39:1681–95. doi: 10.1038/s41388-019-1090-1, PMID: 31705064 PMC8290627

[110] Ueno T Toi M Saji H Muta M Bando H Kuroi K . Significance of macrophage chemoattractant protein-1 in macrophage recruitment, angiogenesis, and survival in human breast cancer. Clin Cancer Res. (2000) 6:3282–9., PMID: 10955814

[111] Zylstra J Whyte GP Beckmann K Pate J Santaolalla A Gervais-Andre L . Exercise prehabilitation during neoadjuvant chemotherapy may enhance tumour regression in oesophageal cancer: results from a prospective non-randomised trial. Br J Sports Med. (2022) 56:402–9. doi: 10.1136/bjsports-2021-104243, PMID: 35105604

