# Supplementary material for: Effects of exercise on angiogenesis biomarkers in cancer patients: a systematic review and meta-analysis
Source: Front Immunol. 2026 Jan 9;16:1705472. doi: 10.3389/fimmu.2025.1705472 (PMC12827781; doi:10.3389/fimmu.2025.1705472)
Supplement: Supplementary file 1 [file DataSheet1.docx]

**Supplementary information 1. PRISMA 2020 checklist.**

| **Section and Topic** | **Item #** | **Checklist item** | **Location where item is reported** |
| --- | --- | --- | --- |
| **TITLE** | | |  |
| Title | 1 | Identify the report as a systematic review. | 1 |
| **ABSTRACT** | | |  |
| Abstract | 2 | See the PRISMA 2020 for Abstracts checklist. | 2 |
| **INTRODUCTION** | | |  |
| Rationale | 3 | Describe the rationale for the review in the context of existing knowledge. | 4 |
| Objectives | 4 | Provide an explicit statement of the objective(s) or question(s) the review addresses. | 4 |
| **METHODS** | | |  |
| Eligibility criteria | 5 | Specify the inclusion and exclusion criteria for the review and how studies were grouped for the syntheses. | 6 |
| Information sources | 6 | Specify all databases, registers, websites, organisations, reference lists and other sources searched or consulted to identify studies. Specify the date when each source was last searched or consulted. | 6 |
| Search strategy | 7 | Present the full search strategies for all databases, registers and websites, including any filters and limits used. | 6 |
| Selection process | 8 | Specify the methods used to decide whether a study met the inclusion criteria of the review, including how many reviewers screened each record and each report retrieved, whether they worked independently, and if applicable, details of automation tools used in the process. | 7 |
| Data collection process | 9 | Specify the methods used to collect data from reports, including how many reviewers collected data from each report, whether they worked independently, any processes for obtaining or confirming data from study investigators, and if applicable, details of automation tools used in the process. | 7 |
| Data items | 10a | List and define all outcomes for which data were sought. Specify whether all results that were compatible with each outcome domain in each study were sought (e.g. for all measures, time points, analyses), and if not, the methods used to decide which results to collect. | 7 |
|  | 10b | List and define all other variables for which data were sought (e.g. participant and intervention characteristics, funding sources). Describe any assumptions made about any missing or unclear information. | 7 |
| Study risk of bias assessment | 11 | Specify the methods used to assess risk of bias in the included studies, including details of the tool(s) used, how many reviewers assessed each study and whether they worked independently, and if applicable, details of automation tools used in the process. | 7 |
| Effect measures | 12 | Specify for each outcome the effect measure(s) (e.g. risk ratio, mean difference) used in the synthesis or presentation of results. | 8 |
| Synthesis methods | 13a | Describe the processes used to decide which studies were eligible for each synthesis (e.g. tabulating the study intervention characteristics and comparing against the planned groups for each synthesis (item #5)). | 8 |
|  | 13b | Describe any methods required to prepare the data for presentation or synthesis, such as handling of missing summary statistics, or data conversions. | 8 |
|  | 13c | Describe any methods used to tabulate or visually display results of individual studies and syntheses. | 8 |
|  | 13d | Describe any methods used to synthesize results and provide a rationale for the choice(s). If meta-analysis was performed, describe the model(s), method(s) to identify the presence and extent of statistical heterogeneity, and software package(s) used. | 8 |
|  | 13e | Describe any methods used to explore possible causes of heterogeneity among study results (e.g. subgroup analysis, meta-regression). | 8 |
|  | 13f | Describe any sensitivity analyses conducted to assess robustness of the synthesized results. | 8 |
| Reporting bias assessment | 14 | Describe any methods used to assess risk of bias due to missing results in a synthesis (arising from reporting biases). | 7 |
| Certainty assessment | 15 | Describe any methods used to assess certainty (or confidence) in the body of evidence for an outcome. | 7 |
| **RESULTS** | | |  |
| Study selection | 16a | Describe the results of the search and selection process, from the number of records identified in the search to the number of studies included in the review, ideally using a flow diagram. | 9 |
|  | 16b | Cite studies that might appear to meet the inclusion criteria, but which were excluded, and explain why they were excluded. | 9 |
| Study characteristics | 17 | Cite each included study and present its characteristics. | 9 |
| Risk of bias in studies | 18 | Present assessments of risk of bias for each included study. | 12 |
| Results of individual studies | 19 | For all outcomes, present, for each study: (a) summary statistics for each group (where appropriate) and (b) an effect estimate and its precision (e.g. confidence/credible interval), ideally using structured tables or plots. | 9 |
| Results of syntheses | 20a | For each synthesis, briefly summarise the characteristics and risk of bias among contributing studies. | 9-11 |
|  | 20b | Present results of all statistical syntheses conducted. If meta-analysis was done, present for each the summary estimate and its precision (e.g. confidence/credible interval) and measures of statistical heterogeneity. If comparing groups, describe the direction of the effect. | 9-11 |
|  | 20c | Present results of all investigations of possible causes of heterogeneity among study results. | 9-11 |
|  | 20d | Present results of all sensitivity analyses conducted to assess the robustness of the synthesized results. | 9-11 |
| Reporting biases | 21 | Present assessments of risk of bias due to missing results (arising from reporting biases) for each synthesis assessed. | 12 |
| Certainty of evidence | 22 | Present assessments of certainty (or confidence) in the body of evidence for each outcome assessed. | 12 |
| **DISCUSSION** | | |  |
| Discussion | 23a | Provide a general interpretation of the results in the context of other evidence. | 13 |
|  | 23b | Discuss any limitations of the evidence included in the review. | 19 |
|  | 23c | Discuss any limitations of the review processes used. | 20 |
|  | 23d | Discuss implications of the results for practice, policy, and future research. | 20 |
| **OTHER INFORMATION** | | |  |
| Registration and protocol | 24a | Provide registration information for the review, including register name and registration number, or state that the review was not registered. | 6 |
|  | 24b | Indicate where the review protocol can be accessed, or state that a protocol was not prepared. | 6 |
|  | 24c | Describe and explain any amendments to information provided at registration or in the protocol. | 6 |
| Support | 25 | Describe sources of financial or non-financial support for the review, and the role of the funders or sponsors in the review. | 31 |
| Competing interests | 26 | Declare any competing interests of review authors. | 31 |
| Availability of data, code and other materials | 27 | Report which of the following are publicly available and where they can be found: template data collection forms; data extracted from included studies; data used for all analyses; analytic code; any other materials used in the review. | 31 |

*From:*  Page MJ, McKenzie JE, Bossuyt PM, Boutron I, Hoffmann TC, Mulrow CD, et al. The PRISMA 2020 statement: an updated guideline for reporting systematic reviews. BMJ 2021;372:n71. doi: 10.1136/bmj.n71. This work is licensed under CC BY 4.0. To view a copy of this license, visit

**Supplementary information 2. Search Strategy.**

| Database | Search Strategy |
| --- | --- |
| Pubmed  n=569 | #1 "Exercise"[MeSH Terms] OR "exercise therapy"[MeSH Terms] OR "physical exercise"[Title/Abstract] OR "physical exercises"[Title/Abstract] OR "physical activity"[Title/Abstract] OR "activities physical"[Title/Abstract] OR "activity physical"[Title/Abstract] OR "physical activities"[Title/Abstract] OR "aerobic exercise"[Title/Abstract] OR "aerobic exercises"[Title/Abstract] OR "exercises aerobic"[Title/Abstract] OR "isometric exercise"[Title/Abstract] OR "isometric exercises"[Title/Abstract] OR "acute exercise"[Title/Abstract] OR "exercise training"[Title/Abstract] OR "exercise therapy"[MeSH Terms] OR "rehabilitation exercise"[Title/Abstract] OR "exercise rehabilitation"[Title/Abstract] OR "exercises rehabilitation"[Title/Abstract] OR "resistance training"[MeSH Terms] OR "strength training"[Title/Abstract] OR (("Weight-Lifting"[MeSH Terms] OR ("weight"[All Fields] AND "lifting"[All Fields]) OR "Weight-Lifting"[All Fields]) AND "strengthening program"[Title/Abstract]) OR "weight lifting exercise program"[Title/Abstract] OR "endurance training"[MeSH Terms] OR "high intensity interval training"[MeSH Terms] OR "circuit based exercise"[MeSH Terms] OR "Pilates"[Title/Abstract] OR "Walking"[Title/Abstract]  #2 "Cancer"[Title/Abstract] OR "tumor"[Title/Abstract] OR "neoplasm"[Title/Abstract] OR "oncology"[Title/Abstract] OR "chemotherapy"[Title/Abstract] OR "radiotherapy"[Title/Abstract] OR "cancer survivors"[MeSH Terms] OR "cancer survivor"[Title/Abstract] OR "survivors cancer"[Title/Abstract] OR "long term cancer survivor"[Title/Abstract] OR "long term cancer survivors"[Title/Abstract] OR "cancer survivorship"[Title/Abstract] OR "neoplasms"[MeSH Terms]  #3 "Biomarkers"[MeSH Terms] OR "Biomarkers"[Title/Abstract] OR "blood biomarkers"[Title/Abstract] OR "angiogenesis"[MeSH Terms] OR "angiogenesis"[Title/Abstract] OR ("vascular endothelial growth factor"[Title/Abstract] OR "VEGF"[Title/Abstract] OR "VEGF-A"[Title/Abstract] OR "VEGF-C"[Title/Abstract] OR "VEGFR"[Title/Abstract] OR "VEGFR-2"[Title/Abstract]) OR ("angiopoietin"[Title/Abstract] OR "Ang-1"[Title/Abstract] OR "Ang-2"[Title/Abstract] OR "Tie2"[Title/Abstract]) OR ("MMP-2"[Title/Abstract] OR "MMP-9"[Title/Abstract] OR "matrix metalloproteinase"[Title/Abstract]) OR ("ICAM-1"[Title/Abstract] OR "VCAM-1"[Title/Abstract] OR "E-selectin"[Title/Abstract] OR "adhesion molecule"[Title/Abstract]) OR ("TGF-beta"[Title/Abstract] OR "TGF-β1"[Title/Abstract]) OR ("HIF-1α"[Title/Abstract] OR "hypoxia-inducible factor"[Title/Abstract]) OR ("PDGF"[Title/Abstract] OR "platelet-derived growth factor"[Title/Abstract]) OR ("bFGF"[Title/Abstract] OR "basic fibroblast growth factor"[Title/Abstract]) OR ("SOCS3"[Title/Abstract]) OR ("IL-8"[Title/Abstract] OR "CXCL8"[Title/Abstract]) OR ("SDF-1"[Title/Abstract] OR "CXCL12"[Title/Abstract]) OR ("CD105"[Title/Abstract] OR "Endoglin"[Title/Abstract]) OR ("eNOS"[Title/Abstract] OR "endothelial nitric oxide synthase"[Title/Abstract]) OR ("circulating biomarkers"[Title/Abstract] OR "angiogenesis-related factors"[Title/Abstract])  #4 "randomized controlled trial"[Publication Type] OR "randomized"[Title/Abstract] OR "placebo"[Title/Abstract] OR "RCT"[Title/Abstract]  #5 #1 AND #2 AND #3 AND #4 |
| Embase  n=1010 | #1. ('exercise'/exp OR 'physical exercise':ti,ab OR 'physical exercises':ti,ab OR 'physical activity':ti,ab OR 'activities, physical':ti,ab OR 'activity, physical':ti,ab OR 'physical activities':ti,ab OR 'aerobic exercise':ti,ab OR 'isometric exercise':ti,ab OR 'acute exercise':ti,ab OR 'exercise training':ti,ab OR 'rehabilitation exercise':ti,ab OR 'exercise therapy'/exp OR 'resistance training'/exp OR 'strength training':ti,ab OR 'weight lifting strengthening program':ti,ab OR 'endurance training'/exp OR 'high intensity interval training'/exp OR 'circuit-based exercise'/exp)  #2. ('cancer'/exp OR 'tumor'/exp OR 'neoplasm'/exp OR 'oncology':ti,ab OR 'chemotherapy':ti,ab OR 'radiotherapy':ti,ab OR 'cancer survivor'/exp OR 'long-term cancer survivor':ti,ab OR 'cancer survivorship':ti,ab)  #3. ('biomarker'/exp OR biomarkers:ti,ab OR 'angiogenesis'/exp OR angiogenesis:ti,ab OR 'vegf':ti,ab OR 'vegf-a':ti,ab OR 'vegf-c':ti,ab OR 'vegfr':ti,ab OR 'vegfr-2':ti,ab OR 'vascular endothelial growth factor'/exp OR 'angiopoietin':ti,ab OR 'ang-1':ti,ab OR 'ang-2':ti,ab OR 'tie2':ti,ab OR 'mmp-2':ti,ab OR 'mmp-9':ti,ab OR 'matrix metalloproteinase':ti,ab OR 'icam-1':ti,ab OR 'vcam-1':ti,ab OR 'e-selectin':ti,ab OR 'adhesion molecule':ti,ab OR 'tgf-beta':ti,ab OR 'tgf-β1':ti,ab OR 'hif-1α':ti,ab OR 'hypoxia inducible factor':ti,ab OR 'pdgf':ti,ab OR 'platelet derived growth factor':ti,ab OR 'fgf-2':ti,ab OR 'basic fibroblast growth factor':ti,ab OR 'socs3':ti,ab OR 'il-8':ti,ab OR 'cxcl8':ti,ab OR 'sdf-1':ti,ab OR 'cxcl12':ti,ab OR 'endoglin':ti,ab OR 'cd105':ti,ab OR 'enos':ti,ab OR 'endothelial nitric oxide synthase':ti,ab OR 'angiogenesis-related factor':ti,ab OR 'circulating angiogenic biomarkers':ti,ab)  #4. ('randomized controlled trial'/exp OR 'randomized controlled trials':ti,ab OR 'randomized trial':ti,ab OR 'controlled clinical trial'/exp OR 'controlled trial':ti,ab OR 'random allocation':ti,ab OR 'double-blind method'/exp OR 'single-blind method'/exp OR 'placebo'/exp OR 'placebo-controlled':ti,ab OR 'randomized':ti,ab OR 'clinical trial':ti,ab OR 'trial protocol':ti,ab OR 'double blind':ti,ab OR 'single blind':ti,ab OR 'crossover trial'/exp OR 'parallel group':ti,ab OR 'random assignment':ti,ab OR 'multicenter study'/exp OR 'multicenter trial':ti,ab OR 'randomization'/exp OR 'randomized study':ti,ab OR 'randomised':ti,ab OR 'randomised controlled trial':ti,ab OR 'sham control':ti,ab OR 'placebo effect':ti,ab OR 'placebo response':ti,ab OR 'control group'/exp OR 'trial design'/exp OR 'intervention study'/exp)  #5. #1 AND #2 AND #3 AND #4 |
| Web of Science  n=272 | #1 TS=("exercise" OR "physical exercise" OR "physical activity" OR "aerobic exercise" OR "resistance training" OR "strength training" OR "exercise training" OR "rehabilitation exercise" OR "isometric exercise" OR "acute exercise" OR "endurance training" OR "high intensity interval training" OR "circuit-based exercise")  #2 TS=("cancer" OR "tumor" OR "neoplasm" OR "oncology" OR "chemotherapy" OR "radiotherapy" OR "cancer survivor" OR "long-term cancer survivor" OR "cancer survivorship")  #3 TS=("angiogenesis" OR "vascular endothelial growth factor" OR "VEGF" OR "VEGF-A" OR "VEGF-C" OR "VEGFR" OR "angiopoietin" OR "Ang-1" OR "Ang-2" OR "Tie2" OR "MMP-2" OR "MMP-9" OR "matrix metalloproteinase" OR "ICAM-1" OR "VCAM-1" OR "E-selectin" OR "adhesion molecule" OR "TGF-beta" OR "TGF-β1" OR "HIF-1α" OR "hypoxia-inducible factor" OR "PDGF" OR "platelet-derived growth factor" OR "bFGF" OR "basic fibroblast growth factor" OR "SOCS3" OR "IL-8" OR "CXCL8" OR "SDF-1" OR "CXCL12" OR "Endoglin" OR "CD105" OR "eNOS" OR "endothelial nitric oxide synthase" OR "angiogenesis-related factors" OR "circulating angiogenic biomarkers" OR "blood biomarkers")  #4 TS=("randomized controlled trial" OR "randomized trial" OR "controlled clinical trial" OR "controlled trial" OR "random allocation" OR "double-blind method" OR "single-blind method" OR "placebo" OR "placebo-controlled" OR "randomized" OR "clinical trial" OR "trial protocol" OR "double blind" OR "single blind" OR "crossover trial" OR "parallel group" OR "random assignment" OR "multicenter study" OR "multicenter trial" OR "randomization" OR "randomized study" OR "randomised" OR "randomised controlled trial" OR "sham control" OR "placebo effect" OR "placebo response" OR "control group" OR "trial design" OR "intervention study")  #5 #1 AND #2 AND #3 AND #4 |
| Cochrane library  n=398 | #1 ("exercise" OR "physical exercise" OR "physical exercises" OR "physical activity" OR "aerobic exercise" OR "isometric exercise" OR "acute exercise" OR "exercise training" OR "rehabilitation exercise" OR "exercise therapy" OR "resistance training" OR "strength training" OR "weight lifting" OR "endurance training" OR "high intensity interval training" OR "circuit-based exercise")  #2 ("cancer" OR "tumor" OR "neoplasm" OR "oncology" OR "chemotherapy" OR "radiotherapy" OR "cancer survivor" OR "long-term cancer survivor" OR "cancer survivorship")  #3 ("angiogenesis" OR "vascular endothelial growth factor" OR "VEGF" OR "VEGF-A" OR "VEGF-C" OR "VEGFR" OR "angiopoietin" OR "Ang-1" OR "Ang-2" OR "Tie2" OR "MMP-2" OR "MMP-9" OR "matrix metalloproteinase" OR "ICAM-1" OR "VCAM-1" OR "E-selectin" OR "adhesion molecule" OR "TGF-beta" OR "TGF-β1" OR "HIF-1α" OR "hypoxia-inducible factor" OR "PDGF" OR "platelet-derived growth factor" OR "bFGF" OR "basic fibroblast growth factor" OR "SOCS3" OR "IL-8" OR "CXCL8" OR "SDF-1" OR "CXCL12" OR "Endoglin" OR "CD105" OR "eNOS" OR "endothelial nitric oxide synthase" OR "angiogenesis-related factor" OR "circulating angiogenic biomarkers" OR "blood biomarker")  #4 ("randomized controlled trial" OR "randomized controlled trials" OR "randomized trial" OR "controlled clinical trial" OR "controlled trial" OR "random allocation" OR "double-blind method" OR "single-blind method" OR "placebo" OR "placebo-controlled" OR "randomized" OR "clinical trial" OR "trial protocol" OR "double blind" OR "single blind" OR "crossover trial" OR "parallel group" OR "random assignment" OR "multicenter study" OR "multicenter trial" OR "randomization" OR "randomized study" OR "randomised" OR "sham control" OR "placebo effect" OR "placebo response" OR "control group" OR "trial design" OR "intervention study")  #5 #1 AND #2 AND #3 AND #4 |
| EBSCOhost  n=98 | #1. (MH "Exercise" OR TI "physical exercise" OR AB "physical exercise" OR TI "physical exercises" OR AB "physical exercises" OR TI "physical activity" OR AB "physical activity" OR TI "activities, physical" OR AB "activities, physical" OR TI "aerobic exercise" OR AB "aerobic exercise" OR TI "isometric exercise" OR AB "isometric exercise" OR TI "acute exercise" OR AB "acute exercise" OR TI "exercise training" OR AB "exercise training" OR TI "rehabilitation exercise" OR AB "rehabilitation exercise" OR (MH "Exercise Therapy") OR (MH "Resistance Training") OR TI "strength training" OR AB "strength training" OR TI "weight lifting strengthening program" OR AB "weight lifting strengthening program" OR (MH "Endurance Training") OR (MH "High Intensity Interval Training") OR (MH "Circuit Training"))  #2. (MH "Neoplasms" OR TI "cancer" OR AB "cancer" OR TI "tumor" OR AB "tumor" OR TI "neoplasm" OR AB "neoplasm" OR TI "oncology" OR AB "oncology" OR TI "chemotherapy" OR AB "chemotherapy" OR TI "radiotherapy" OR AB "radiotherapy" OR (MH "Cancer Survivors") OR TI "long-term cancer survivor" OR AB "long-term cancer survivor" OR TI "cancer survivorship" OR AB "cancer survivorship")  #3. (MH "Biomarkers" OR TI "biomarker" OR AB "biomarker" OR TI "blood biomarker" OR AB "blood biomarker" OR TI "angiogenesis" OR AB "angiogenesis" OR TI "vascular endothelial growth factor" OR AB "vascular endothelial growth factor" OR TI "VEGF" OR AB "VEGF" OR TI "VEGF-A" OR AB "VEGF-A" OR TI "VEGFR" OR AB "VEGFR" OR TI "angiopoietin" OR AB "angiopoietin" OR TI "Ang-1" OR AB "Ang-1" OR TI "Ang-2" OR AB "Ang-2" OR TI "Tie2" OR AB "Tie2" OR TI "MMP-2" OR AB "MMP-2" OR TI "MMP-9" OR AB "MMP-9" OR TI "matrix metalloproteinase" OR AB "matrix metalloproteinase" OR TI "ICAM-1" OR AB "ICAM-1" OR TI "VCAM-1" OR AB "VCAM-1" OR TI "E-selectin" OR AB "E-selectin" OR TI "adhesion molecule" OR AB "adhesion molecule" OR TI "TGF-beta" OR AB "TGF-beta" OR TI "TGF-β1" OR AB "TGF-β1" OR TI "HIF-1α" OR AB "HIF-1α" OR TI "hypoxia-inducible factor" OR AB "hypoxia-inducible factor" OR TI "PDGF" OR AB "PDGF" OR TI "platelet-derived growth factor" OR AB "platelet-derived growth factor" OR TI "bFGF" OR AB "bFGF" OR TI "basic fibroblast growth factor" OR AB "basic fibroblast growth factor" OR TI "SOCS3" OR AB "SOCS3" OR TI "IL-8" OR AB "IL-8" OR TI "CXCL8" OR AB "CXCL8" OR TI "SDF-1" OR AB "SDF-1" OR TI "CXCL12" OR AB "CXCL12" OR TI "Endoglin" OR AB "Endoglin" OR TI "CD105" OR AB "CD105" OR TI "eNOS" OR AB "eNOS" OR TI "endothelial nitric oxide synthase" OR AB "endothelial nitric oxide synthase" OR TI "angiogenesis-related factors" OR AB "angiogenesis-related factors" OR TI "circulating angiogenic biomarkers" OR AB "circulating angiogenic biomarkers")  #4. (MH "Randomized Controlled Trials" OR TI "randomized controlled trials" OR AB "randomized controlled trials" OR TI "randomized trial" OR AB "randomized trial" OR (MH "Controlled Clinical Trials") OR TI "controlled trial" OR AB "controlled trial" OR TI "random allocation" OR AB "random allocation" OR (MH "Double-Blind Method") OR (MH "Single-Blind Method") OR (MH "Placebos") OR TI "placebo" OR AB "placebo" OR TI "randomized" OR AB "randomized" OR TI "clinical trial" OR AB "clinical trial" OR TI "trial protocol" OR AB "trial protocol" OR TI "double blind" OR AB "double blind" OR TI "single blind" OR AB "single blind" OR (MH "Crossover Design") OR TI "parallel group" OR AB "parallel group" OR TI "random assignment" OR AB "random assignment" OR (MH "Multicenter Studies") OR TI "multicenter trial" OR AB "multicenter trial" OR TI "randomization" OR AB "randomization" OR TI "randomised" OR AB "randomised" OR TI "sham control" OR AB "sham control" OR TI "placebo effect" OR AB "placebo effect" OR TI "control group" OR AB "control group" OR (MH "Trial Design") OR (MH "Intervention Studies"))  #5. #1 AND #2 AND #3 AND #4 |

**Supplementary information 3. Information on RCTs Included in the Meta-Analysis.**

| No | RCT Information | | Patient background information | | | | | Exercise prescription information | | | | | Outcome | |
| --- | --- | --- | --- | --- | --- | --- | --- | --- | --- | --- | --- | --- | --- | --- |
| . | Study | Study registration number | Number of patients | Cancer type | Stage | Comorbidities | Treatment timing | Type | Duration (week) | Exercise prescription parameters | Supervision | Other Intervention | Outcome indicators included in the meta-analysis | Only the effect size was calculated for the outcome |
| 1 | Sturgeon2023 | NCT01515124 | 255 | BC | I-III | Not reported | Survivor | AE+RT | 52 | AE: 180 min/week, moderate-intensity (MI).  RT: 9 RT, 10 reps/1set, 2 times/week. | Yes | Calorie restriction | ICAM-1, VCAM-1 |  |
|  |  |  |  |  |  |  |  | AE+RT | 52 | AE: 180 min/week, moderate-intensity.  RT: 9 RT, 10 reps/1set, 2 times/week. | Yes | No | ICAM-1, VCAM-1 |  |
| 2 | Cartmel2023 | NCT02107066 | 104 | OC | I-IV | Not reported | Survivor | AE | 24 | 150 min/week MI. | No | No | VEGF |  |
| 3 | Moon2022 | NCT03397030 | 27 | PCa | Newly diagnosed patients | Not reported | Before | AE+RT | 24 | AE: 150 min/week, 30 min/time, 40-60% HRR. RT: 3 RT, 15 reps/1set, 3sets, 3 times/week.. | No | No | VEGF |  |
| 4 | Kennedy2021 | NCT03314311 | 37 | GC | I-III | Not reported | Survivor | AE+RT | 12 | AE: 45-60% HRR. RT: 17 reps/1set, 6sets. | No | No | VEGF, ICAM-1, VCAM-1, MCP-1, VEGF-C | FGF, PIGF, VEGFR-1, VEGF-D |
| 5 | Hiensch2021 | NCT02522260 | 57 | BC | I-III | Not reported | During | AE+RT | 16 | RT: 80% 1RM, 8-12 reps/set,2-3 sets. HIIT: 3×3 min, 6-18 RPE,1 min recovery.  AE+RT: 2 times/week. | Yes | No | VEGF-A, VEGF-C | ANG-1, ANG-2, PIGF, HGF, EGF, PDGF subunit B, VEGFR-2 |
|  |  |  |  |  |  |  |  | AE | 16 | AE: 20 min, 13-15 RPE. HITT: 3×3min, 16-18 RPE, 1 min recovery.  AE+HIIT: 2 times/week. | Yes | No | VEGF-A, VEGF-C | ANG-1, ANG-2, PIGF, HGF, EGF, PDGF subunit B, VEGFR-2 |
| 6 | Demark-Wahnefried2020 | NCT02224807 | 32 | BC | 0-II | BMI≥25 | Before | AE | 4 | 30 min/day. | Yes | Calorie restriction | VEGF-C | FGF |
| 7 | Brown2018 | NCT02250053 | 39 | CRC | I-III | Not reported | Survivor | AE | 24 | 150 min/week, 50−70% HRR. | No | No | ICAM-1, VCAM-1 |  |
|  |  |  |  |  |  |  |  | AE | 24 | 300 min/week, 50−70% HRR. | No | No | ICAM-1, VCAM-1 |  |
| 8 | Demark-Wahnefried2017 | NCT01886677 | 40 | PCa | Newly diagnosed patients | Not reported | Before | AE | 6 | 30 min/day | No | Calorie restriction | VEGF |  |
| 9 | Glass2015 |  | 44 | Mixed | Not reported | Few participants had heart disease. | During | AE | 12 | 3 times/week, 45 min/time, 55-100% VO_2peak_. | Yes | No | VEGF |  |
| 10 | LeeJones2013 | NCT00405678 | 20 | BC | II-III | Few participants had hypertension, hyperlipidemia, arthritis, or diabetes | During | AE | 12 | 3 times/week, 40 min/time, 55-100% VO_2max_ | Yes | No |  | VEGFR-2 |
| 11 | Ergun2013 |  | 60 | BC | Not reported | Not reported | Survivor | AE+RT | 12 | AE: 30 min.  RT: 15 min.  AE+RT: 45 min/time. | Yes | No | VEGF, MCP-1 | ANG, PDGF |
|  |  |  |  |  |  |  |  | AE | 12 | 30 min/time | Yes | No | VEGF, MCP-1 | ANG |
| 12 | Pakiz2011 |  | 68 | BC | I-III | Not reported | Survivor | AE | 16 | 60 min/day | No | Calorie restriction | VEGF |  |
| 13 | Gómez2011 |  | 18 | BC | I-II | Not reported | Survivor | AE+RT | 8 | AE+RT: 90 min/time.  5 min warm-up; AE: 30 min, 80% HRR;  RT: 12-15 RM, 8-10 reps/set, 2 sets; 5 min cool-down. | Yes | No | VEGF, ICAM-1, VCAM-1, MCP-1 | FGF, PDGF-BB |

**Supplementary information 4.Overall effect size and sensitivity analyses**

Table S1. RVE model parameters without moderator variables

|  | ES | SE | t | dfs | prob | CI.L | CI.U | sig | I² |
| --- | --- | --- | --- | --- | --- | --- | --- | --- | --- |
| VEGF | -0.14 | 0.14 | -0.97 | 7.49 | 0.36 | -0.47 | 0.19 |  | 45.04% |
| VEGF-C | 0.07 | 0.06 | 1.12 | 1.87 | 0.38 | -0.22 | 0.37 |  | 0 |
| ICAM-1 | 0.05 | 0.19 | 0.26 | 2.59 | 0.81 | -0.63 | 0.74 |  | 47.52% |
| VCAM-1 | 0.24 | 0.09 | 2.57 | 1.59 | 0.15 | -0.28 | 0.77 |  | 0 |
| MCP-1 | -0.20 | 0.16 | -1.21 | 1.76 | 0.36 | -0.98 | 0.59 |  | 0 |

Table S2. Sensitivity analysis results

| NO. | 1 | 2 | 3 | 4 | 5 | 6 | 7 | 8 | 9 |
| --- | --- | --- | --- | --- | --- | --- | --- | --- | --- |
| rho | 0.1 | 0.2 | 0.3 | 0.4 | 0.5 | 0.6 | 0.7 | 0.8 | 0.9 |
| VEGF | -0.14 | -0.14 | -0.14 | -0.14 | -0.14 | -0.14 | -0.14 | -0.14 | -0.14 |
| VEGF-C | 0.07 | 0.07 | 0.07 | 0.07 | 0.07 | 0.07 | 0.07 | 0.07 | 0.07 |
| ICAM-1 | 0.05 | 0.05 | 0.05 | 0.05 | 0.05 | 0.05 | 0.05 | 0.05 | 0.05 |
| VCAM-1 | 0.24 | 0.24 | 0.24 | 0.24 | 0.24 | 0.24 | 0.24 | 0.24 | 0.24 |
| MCP-1 | -0.20 | -0.20 | -0.20 | -0.20 | -0.20 | -0.20 | -0.20 | -0.20 | -0.20 |


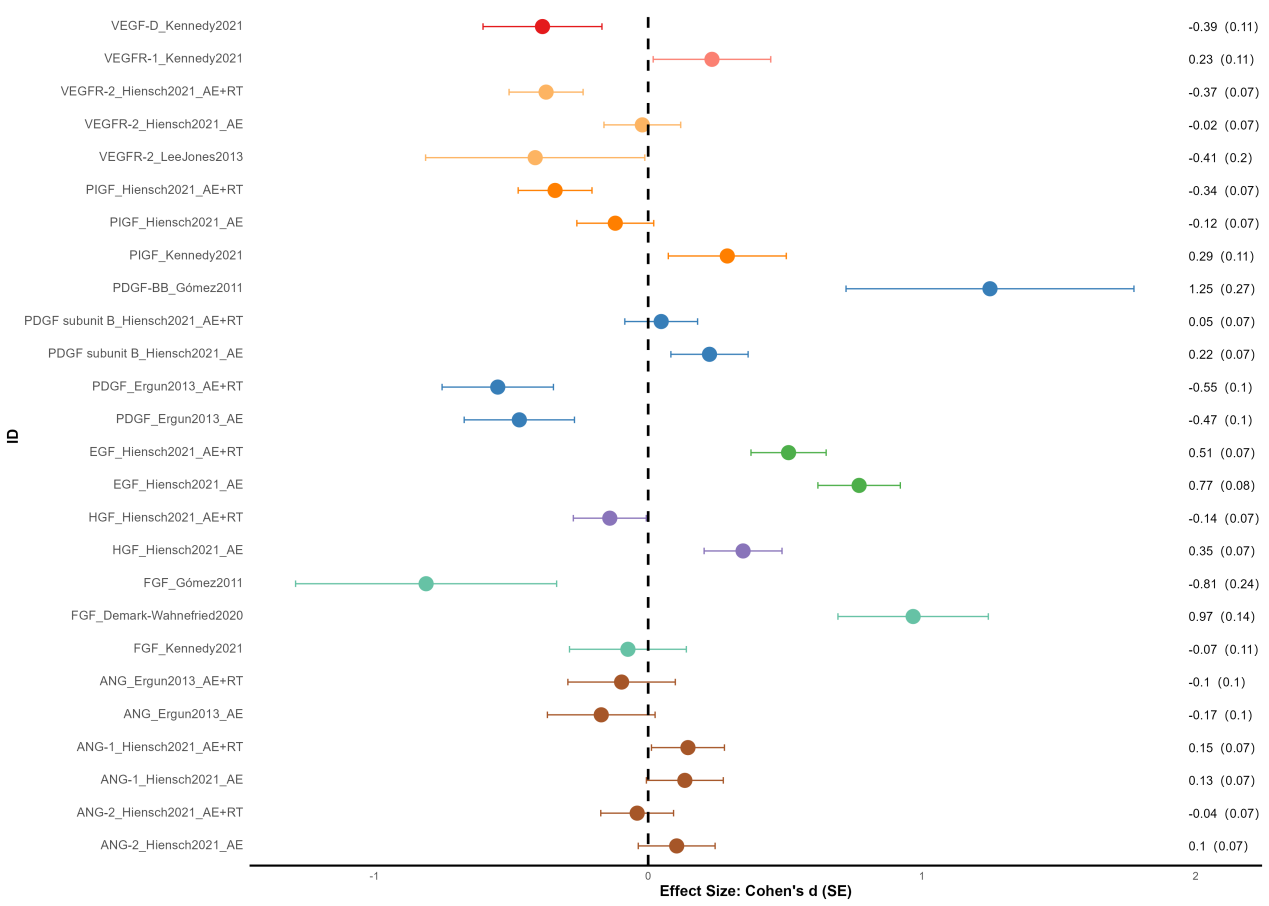


Fig. S1. The effect sizes that were not included in the meta-analysis were summarized.

**Supplementary information 5. Results of meta-regression analysis.**

Table S3. Moderating variable information summary

| NO. | Name | Extraction Strategy |
| --- | --- | --- |
| 1 | EI.Type (AE/RT/PA/AE+RT) | AE is aerobic exercise. RT is resistance exercise. PA is a recommendation for increasing physical activity. |
| 2 | EI Duration | Duration is the duration of the exercise intervention. Only pre- and post-intervention values are included. |
| 3 | Single Exercise Intensity | HRmax/HRR (Heart Rate Reserve): 50%–70% = 3–6 MET, 70%–85% = 6–8 MET, >85% = 8-10 MET.  If the intensity of RT is not clearly marked, it is calculated according to 4.5 MET.  Borg scale: 12–20 = 4–10 MET. |
| 4 | Weekly Exercise Duration | Total time of exercise intervention per week. |
| 5 | Weekly Exercise Volume | Weekly Exercise Volume = Single Exercise Intensity × Weekly Exercise Duration |
| 6 | Supervision (Yes/No) | Yes indicates that the exercise intervention included any form of supervision, whether fully supervised (e.g., in-person or virtual sessions throughout the intervention period) or partially supervised (e.g., supervision provided only during the initial weeks or combined with home-based sessions).  No indicates no supervision, including interventions with only initial demonstrations, home-based programs without regular contact, or those delivered solely through telephone guidance. |
| 7 | Other Intervention (Calorie restriction/No) | No indicates the absence of structured co-interventions. General recommendations such as simple psychological guidance, basic nutritional supplementation, weight loss advice, or the lack of a clearly defined caloric restriction strategy were all classified as No. |
| 8 | Timing (Before/During/Survivor) | Before indicates that the intervention was implemented during the prehabilitation phase or the active surveillance period prior to primary cancer treatment.  During indicates that the intervention was delivered during the course of cancer treatment (e.g., surgery, chemotherapy, radiotherapy, or hormone therapy).  Survivor indicates that the intervention was administered after the completion of all cancer treatments. |
| 9 | Age | Age is the mean age of the RCTs. |
| 10 | BMI | BMI is the mean body mass index of the RCTs. |
| 11 | Cancer Type | BC = Breast cancer;  GC = Gastric cancer;  Mixed = Mixed cancer types;  OC = Ovarian cancer;  PCa = Prostate cancer. |
| 12 | Detection Method | Detection Method refers to the assay technique used for cytokine measurement, such as ELISA, MSD, and others. |

Table S4. Results of univariate regression analysis of VEGF.

| labels | b.r | SE | t | dfs | prob | CI.L | CI.U | sig | I^2^ (%) | R^2^ |
| --- | --- | --- | --- | --- | --- | --- | --- | --- | --- | --- |
| EI.Type | | | | | | | | | | |
| X.Intercept.(AE) | -0.04 | 0.24 | -0.15 | 3.37 | 0.89 | -0.76 | 0.69 |  | 44.4% | 0.03 |
| AE+RT | -0.22 | 0.26 | -0.83 | 7.13 | 0.43 | -0.84 | 0.40 |  |  |  |
| Single.Exercise.Intensity | | | | | | | | | | |
| X.Intercept. | 0.05 | 0.38 | 0.12 | 5.27 | 0.91 | -0.92 | 1.01 |  | 50.45% | -0.24 |
| Single.Exercise.Intensity | -0.03 | 0.06 | -0.53 | 3.95 | 0.63 | -0.19 | 0.13 |  |  |  |
| Weekly.Exercise.Duration | | | | | | | | | | |
| X.Intercept. | -0.41 | 0.31 | -1.30 | 4.32 | 0.26 | -1.25 | 0.44 |  | 46.82% | -0.07 |
| Weekly.Exercise.Duration | 0.00 | 0.00 | 1.12 | 3.27 | 0.34 | 0.00 | 0.00 |  |  |  |
| Weekly.Exercise.Volume | | | | | | | | | | |
| X.Intercept. | -0.32 | 0.38 | -0.85 | 4.75 | 0.44 | -1.30 | 0.66 |  | 48.19% | -0.13 |
| Weekly.Exercise.Volume | 0.00 | 0.00 | 0.47 | 3.62 | 0.67 | 0.00 | 0.00 |  |  |  |
| EI.Duration | | | | | | | | | | |
| X.Intercept. | -0.50 | 0.39 | -1.30 | 4.15 | 0.26 | -1.56 | 0.56 |  | 41.98% | 0.11 |
| EI.Duration | 0.02 | 0.02 | 1.03 | 2.97 | 0.38 | -0.05 | 0.10 |  |  |  |
| Other.Intervention | | | | | | | | | | |
| X.Intercept.(Calorie restriction) | 0.15 | 0.13 | 1.18 | 1.96 | 0.36 | -0.41 | 0.71 |  | 42.04% | 0.11 |
| No | -0.43 | 0.24 | -1.82 | 4.02 | 0.14 | -1.09 | 0.23 |  |  |  |
| Supervision | | | | | | | | | | |
| X.Intercept.(No) | 0.15 | 0.10 | 1.42 | 3.03 | 0.25 | -0.18 | 0.48 |  | 0.65% | 0.99 |
| Yes | -0.60 | 0.24 | -2.52 | 4.99 | 0.05 | -1.20 | 0.01 | * |  |  |
| Timing | | | | | | | | | | |
| X.Intercept.(Before) | -0.17 | 0.23 | -0.73 | 1.00 | 0.60 | -3.09 | 2.75 |  | 47.4% | -0.09 |
| During | -0.23 | 0.42 | -0.54 | 1.97 | 0.65 | -2.08 | 1.63 |  |  |  |
| Survivor | 0.14 | 0.30 | 0.46 | 1.71 | 0.70 | -1.39 | 1.67 |  |  |  |
| Age | | | | | | | | | | |
| X.Intercept. | -0.62 | 1.41 | -0.44 | 2.81 | 0.69 | -5.27 | 4.04 |  | 49.69% | -0.20 |
| Age | 0.01 | 0.02 | 0.34 | 2.39 | 0.76 | -0.08 | 0.10 |  |  |  |
| BMI | | | | | | | | | | |
| X.Intercept. | -2.13 | 1.21 | -1.76 | 3.42 | 0.17 | -5.72 | 1.46 |  | 29.48% | 0.49 |
| BMI | 0.07 | 0.04 | 1.67 | 2.97 | 0.20 | -0.07 | 0.21 |  |  |  |
| Cancer.Type | | | | | | | | | | |
| X.Intercept.(BC) | -0.15 | 0.24 | -0.62 | 2.77 | 0.58 | -0.94 | 0.65 |  | 46.48% | -0.05 |
| GC | 0.13 | 0.24 | 0.55 | 2.77 | 0.62 | -0.66 | 0.92 |  |  |  |
| Mixed | -0.63 | 0.24 | -2.67 | 2.77 | 0.08 | -1.43 | 0.16 | * |  |  |
| OC | 0.41 | 0.24 | 1.72 | 2.77 | 0.19 | -0.38 | 1.20 |  |  |  |
| PCa | -0.02 | 0.33 | -0.06 | 1.91 | 0.96 | -1.51 | 1.47 |  |  |  |
| Detection.Method | | | | | | | | | | |
| X.Intercept.(ELISA) | 0.03 | 0.19 | 0.18 | 2.93 | 0.87 | -0.58 | 0.65 |  | 46.06% | -0.04 |
| Luminex | -0.65 | 0.19 | -3.41 | 2.93 | 0.04 | -1.26 | -0.03 | ** |  |  |
| MS | -0.45 | 0.19 | -2.36 | 2.93 | 0.10 | -1.06 | 0.16 |  |  |  |
| MSD | -0.30 | 0.33 | -0.90 | 4.08 | 0.42 | -1.22 | 0.62 |  |  |  |


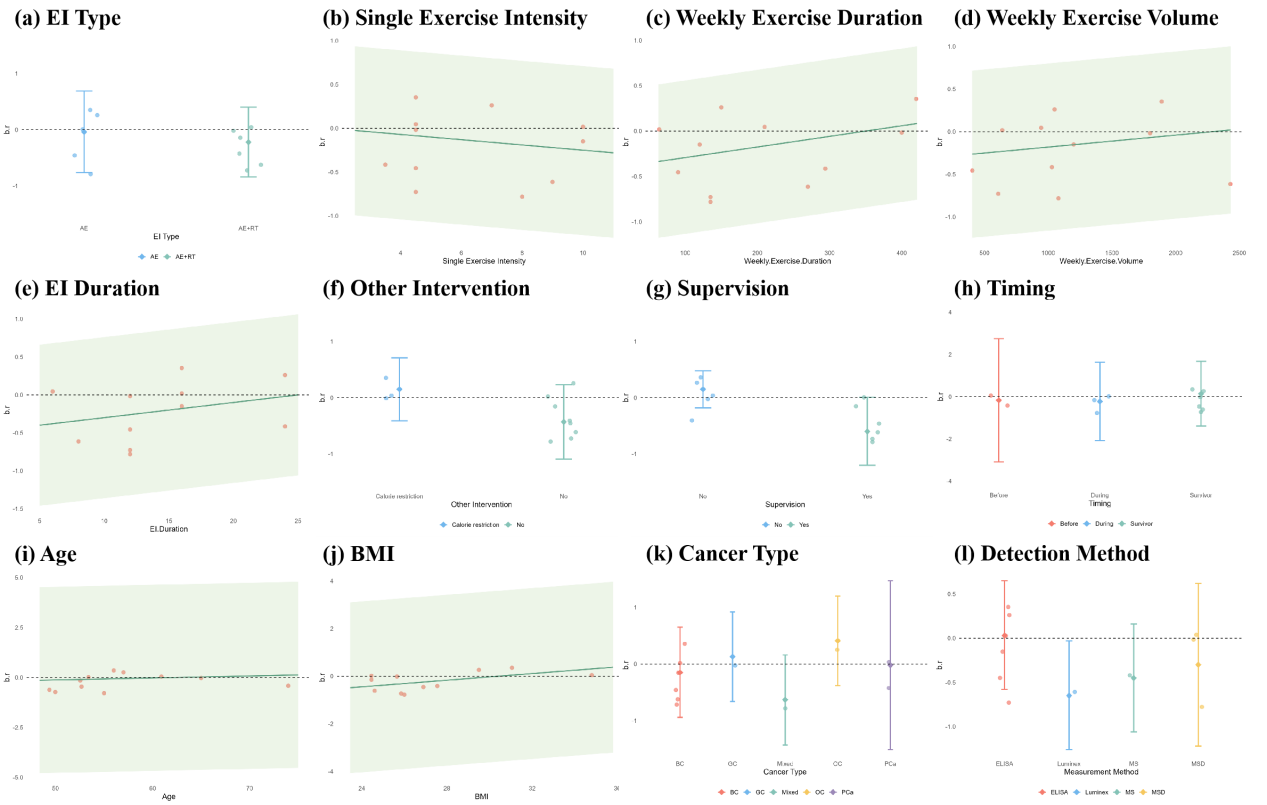


Fig.S2. Univariate regression analysis results of VEGF.

**Supplementary information 6. Risk of bias, publication bias and quality of evidence.**


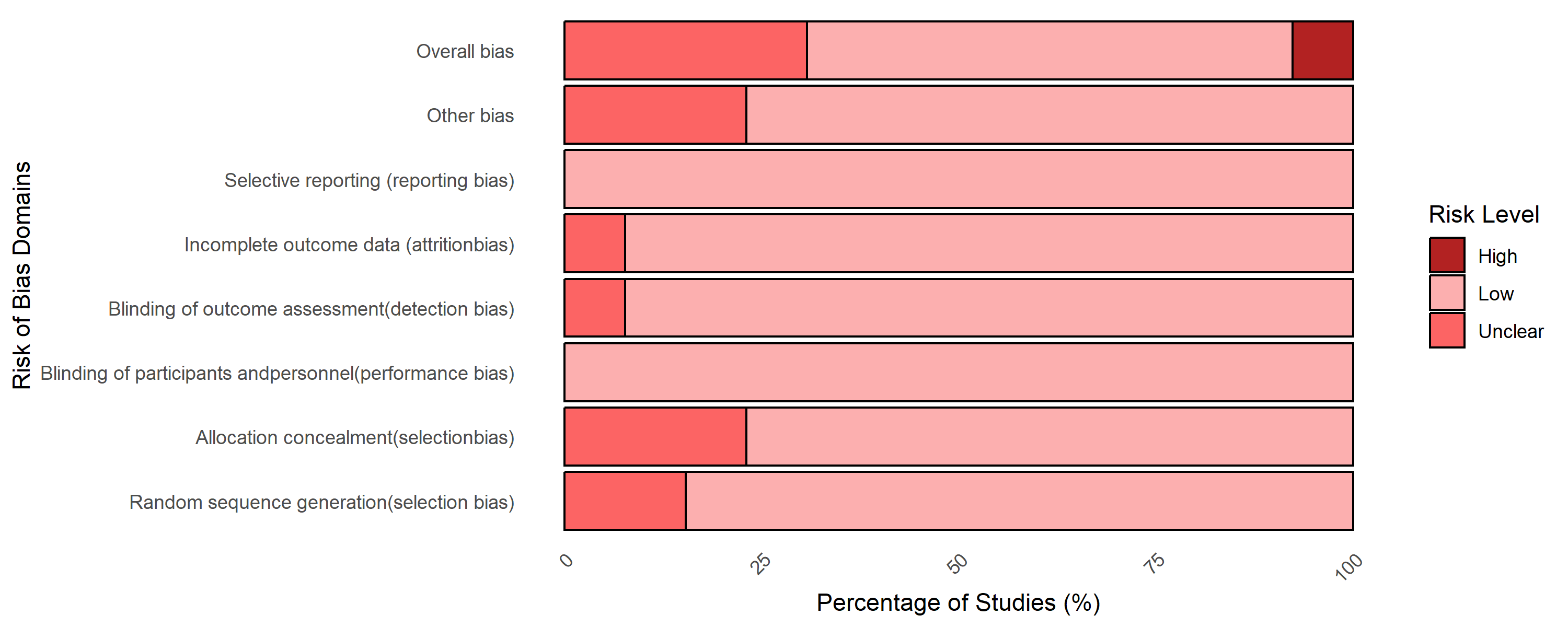


Fig. S3. Summary of risk of bias


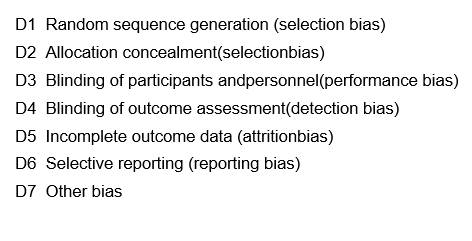

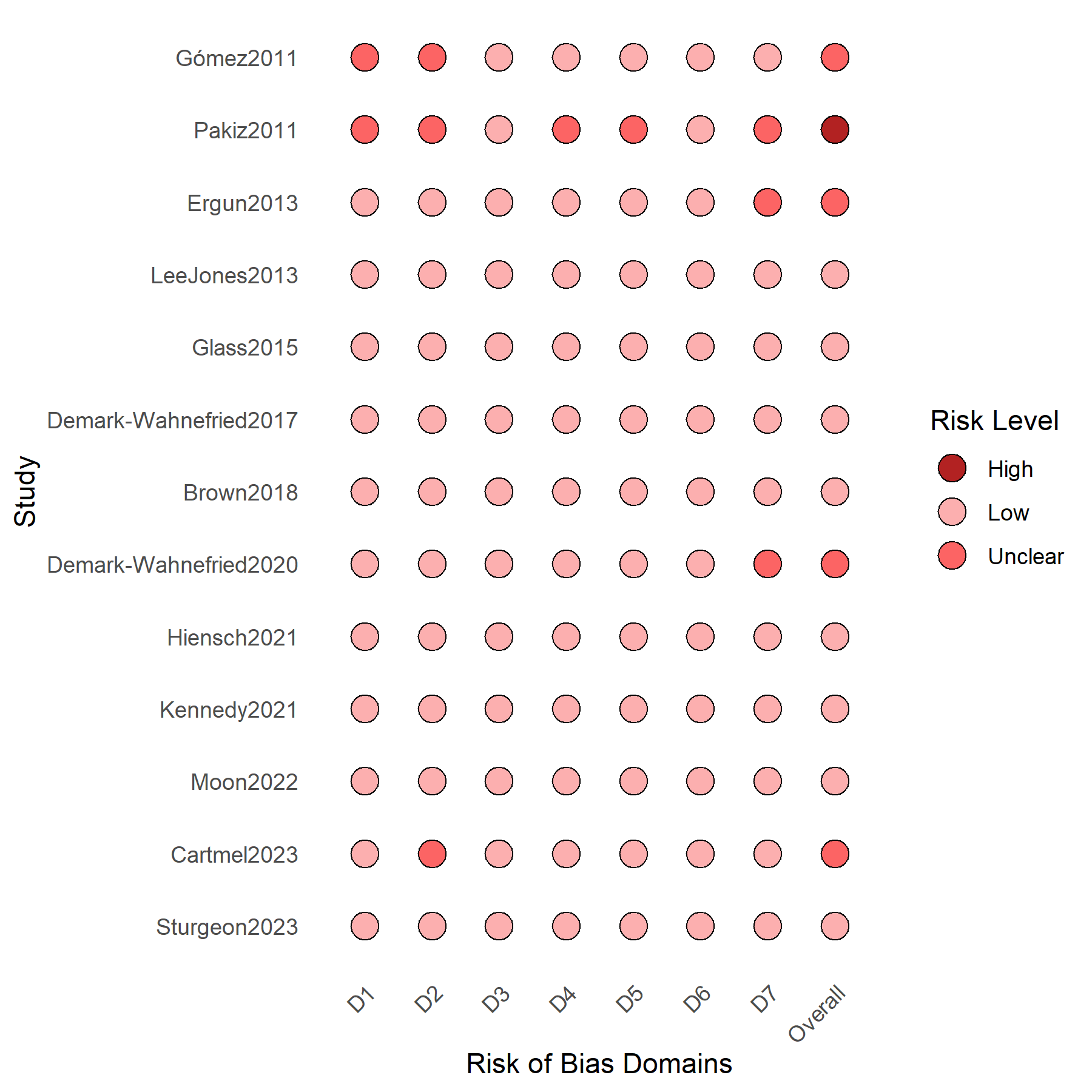


Fig. S4. Details of risk of bias


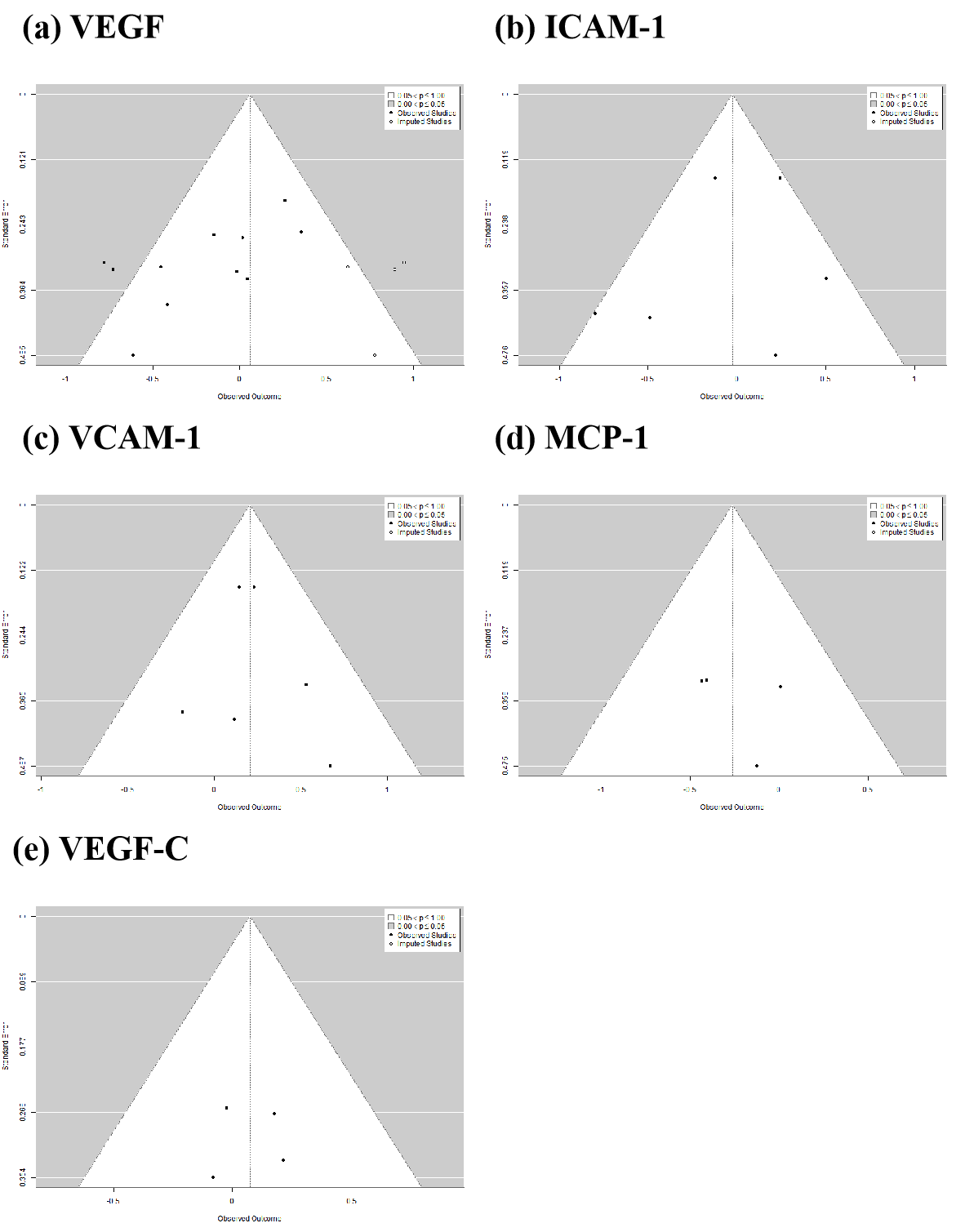


Fig. S5. Funnel plot

Table S5. GRADE rating for effect size

| Outcome | Effect Size | SE | Risk of Bias (ROB2) | Imprecision(p) | Indirectness | Inconsistency (I²) | Publication Bias | GRADE Quality |
| --- | --- | --- | --- | --- | --- | --- | --- | --- |
| VEGF | -0.14 | 0.14 | 1/9 High Risk  3/9 Unclear Risk* | 0.36† | Direct | 45.04%† | Egger’s p > 0.05 | Very Low*‡ |
| ICAM-1 | 0.05 | 0.19 | 1/4 Unclear Risk* | 0.81† | Direct | 47.52%† |  | Very Low*‡ |
| VCAM-1 | 0.24 | 0.09 | 1/4 Unclear Risk* | 0.15† | Direct | 0 |  | Low*† |
| MCP-1 | -0.20 | 0.16 | 2/3 Unclear Risk* | 0.36† | Direct | 0 |  | Low*† |
| VEGF-C | 0.07 | 0.06 | 1/3 Unclear Risk* | 0.38† | Direct | 0 |  | Low*† |

Note: Reasons for downgrading: †Imprecision (1 downgrade), ‡Severe imprecision (2 downgrades), *risk of bias (1 downgrade) , #inconsistency (1 downgrade).
